# Single-cell sequencing technology in renal cancer: insights into tumor biology and clinical application

**DOI:** 10.1186/s40364-025-00811-0

**Published:** 2025-10-01

**Authors:** Hanzhong Zhang, Ying Liu, Wenqiang Liu, Anqi Lin, Yu Fang, Le Qu, Xu Zhang, Peng Luo, Linhui Wang, Aimin Jiang

**Affiliations:** 1 Department of Urology, Changhai Hospital, Naval Medical University (Second Military Medical University), Shanghai, China; 2 Donghai County Peoples Hospital, Jiangnan University Smart Healthcare Joint Laboratory, Donghai County People’s Hospital (Affiliated Kangda College of Nanjing Medical University), Lianyungang, China; 3 Department of Oncology, Zhujiang Hospital, Southern Medical University, Guangzhou, China; 4 Department of Urology, Affiliated Jinling Hospital, Medical School of Nanjing University, Nanjing, China; 5 Department of Urology, Chinese PLA General Hospital, Beijing, China

**Keywords:** Renal cell carcinoma (RCC), Single-cell sequencing (SCS), Single-cell multi-omics, Spatial transcriptomics (ST), Tumor microenvironment (TME), Tumor heterogeneity, Personalized medicine

## Abstract

Renal cancer, particularly clear cell renal cell carcinoma (ccRCC), is characterized by significant intratumoral heterogeneity, which poses challenges for diagnosis and treatment. Single-cell sequencing (SCS) provides unprecedented insights into the cellular landscape of renal cancer, allowing for detailed characterization of tumor heterogeneity at the single-cell level. This review highlights how SCS has been instrumental in elucidating the origins of different renal cancer subtypes, understanding mechanisms of tumor initiation and progression, and dissecting the complex tumor microenvironment (TME). It discusses the identification of novel biomarkers and therapeutic targets, as well as the potential of SCS to inform personalized treatment strategies. The review also explores the integration of SCS with spatial omics technologies, which enhances the understanding of cellular interactions within their spatial context. Moreover, it addresses the challenges and future directions in applying SCS to clinical practice, emphasizing its significance in advancing renal cancer biology and improving clinical interventions.

## Introduction

Renal cancer is a significant global health concern, with a high prevalence among various malignancies. In 2022, there were approximately 434,419 new cases of kidney cancer worldwide [1]. Renal cell carcinoma (RCC) is the most common type of renal cancer, accounting for around 80% of cases. It is a complex disease with diverse histopathological entities, with ccRCC being the most prevalent subtype. The remaining 20% consists of non-clear cell renal cell carcinoma (nccRCC) subtypes, each having unique molecular and cytogenetic profiles that contribute to the heterogeneity of the disease [2]. Recent research has focused on the genetic aspects of RCC, revealing that approximately 6–9% of cases exhibit germline mutations in genes associated with cancer predisposition. However, the actual incidence of such genetic variants may be higher. Understanding these genetic factors is crucial as they could lead to the identification of novel therapeutic targets [3]. Despite advancements in research, the complexity of renal cancer, including its resistance to conventional treatments, remains a major challenge in effective management and treatment [4].

Traditional renal cancer research and diagnostics have been constrained by their inability to fully encompass the heterogeneity of the disease. Bulk sequencing methods, which have been the cornerstone of genetic analysis, have often failed to capture the nuance of cellular heterogeneity characteristic of renal cancer [5, 6]. This limitation hinders a comprehensive understanding of the tumor's molecular landscape, particularly in identifying different subpopulations of cells within a tumor that may respond differently to treatment. There is a need for more precise tools that can uncover the complex interplay of genetic and epigenetic factors [7] that drive renal cancer's progression and resistance to therapy. driving renal cancer progression and therapy resistance. Additionally, traditional methods lack the resolution to identify crucial cell populations that may play pivotal roles in cancer evolution and metastasis. The emergence of SCS technology represents a significant advancement in cancer research. It provides unprecedented insights into the cellular landscape of diseases like renal cancer at a single-cell resolution. Unlike bulk sequencing methods that provide an averaged view, SCS enables the detailed characterization of cellular heterogeneity within tumors. This technology has the potential to identify crucial cell populations driving cancer progression and therapeutic resistance [8–13]. By dissecting the tumor microenvironment (TME) at the single-cell level [14], SCS unveils novel biomarkers and enhances our understanding of the molecular pathways underlying cancer's aggressive behavior [15–20]. The transformative potential of SCS in renal cancer biology lies in its ability to pave the way for targeted therapies and personalized treatment strategies. It can enhance predictive capabilities regarding treatment outcomes, improve patient stratification, and inform clinical management.

This review aims to provide a comprehensive overview of the current state and cutting-edge advancements of SCS technology in renal cancer research. It will highlight the fundamental processes underlying SCS and recent breakthroughs that have advanced our understanding of cellular and molecular intricacies in renal cancer. The review will explore the applications of SCS in renal cancer research and emphasize pivotal findings, including the origins of different renal cancer subtypes, mechanisms of initiation and progression, the comprehensive panorama of the TME, and the complexity of tumor heterogeneity. Furthermore, it will discuss the prospects of SCS in shaping clinical practice, emphasizing its potential to inform personalized treatment strategies, facilitate the development of targeted therapies, and improve prognostication. Ultimately, this review underscores the indispensability of SCS in advancing renal cancer biology and its significance in the quest for more effective and precise clinical interventions.

## Principles and advances in single-cell sequencing technology

### Principles of single-cell sequencing technology

SCS is a cutting-edge methodology that allows for the analysis of individual cells at the molecular level, including gene expression profiles and epigenetic signatures [6]. It employs various techniques to capture, analyze, and process data from single cells, providing insights into the complexity and heterogeneity of cellular behavior under different conditions. SCS has been widely applied in fields such as cancer research, neuroscience, and microbiology. In cancer research, single-cell RNA sequencing (scRNA-seq) has been used to study tumor heterogeneity, cell differentiation, and immune responses [10, 21, 22]. In neuroscience, it aids in identifying neuronal subtypes and functional annotations [23, 24].

**Fig. 1:**
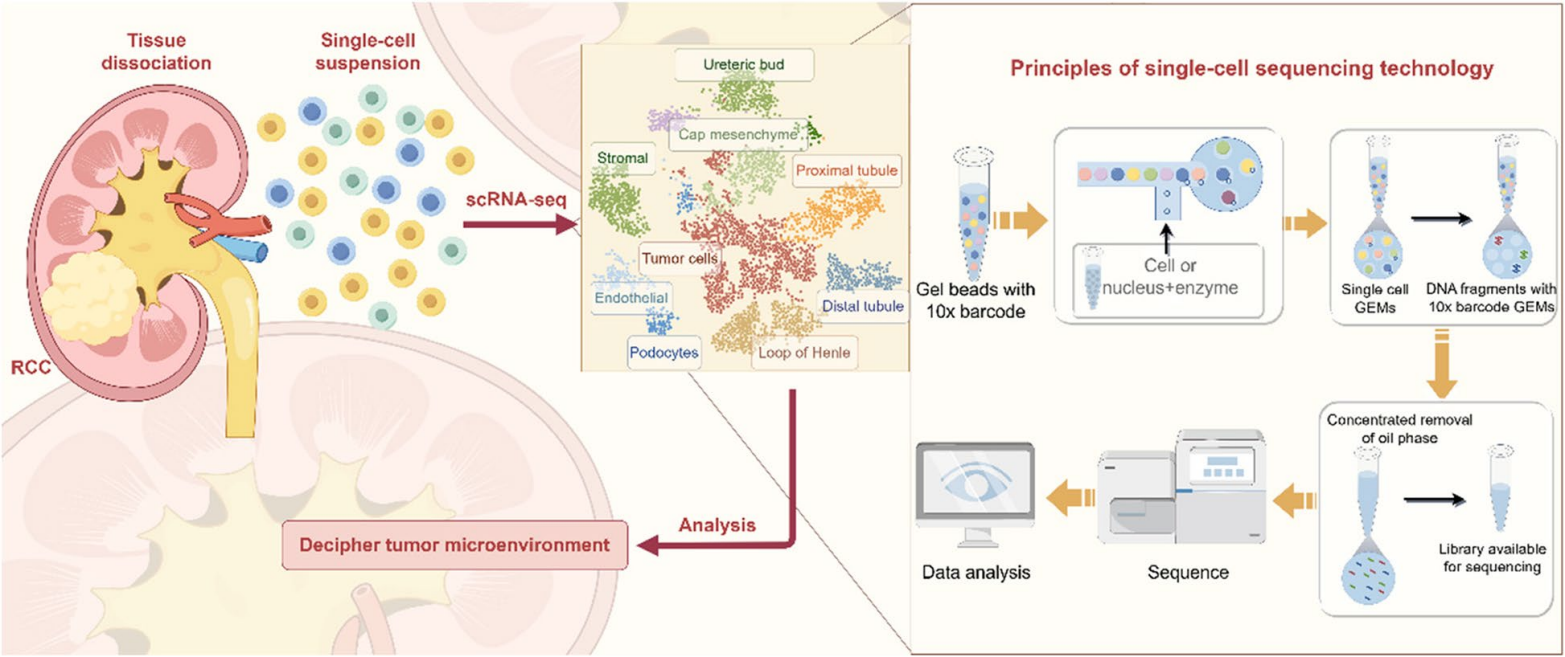
Principles of single-cell sequencing technology. Single-cell sequencing (SCS) involves a series of methodical steps that begin with the isolation of individual cells from heterogeneous tissue samples. Each cell is then barcoded to track its origin, and a library is constructed by converting RNA into complementary DNA (cDNA) through amplification. The subsequent sequencing is followed by a rigorous data analysis phase, which includes noise reduction, duplicate removal, and the segregation of cell types. The culmination of this process is the visualization of the data, providing a detailed map of cellular gene expression, functional status, and heterogeneity. This figure was created based on the tools provided by Figuredraw.com

The first step in SCS involves isolating individual cells from complex tissue samples. Common methods for cell isolation include Fluorescence-Activated Cell Sorting (FACS), microfluidic approaches, and flow cytometry. These techniques efficiently isolate single cells, which is essential for subsequent molecular analysis [25–28]. During the SCS process, each cell is individually captured and barcoded using techniques such as droplet-based methods or in situ barcoding. Barcoding assigns a unique identifier to each cell, facilitating further analysis [29, 30]. RNA is then extracted from each cell, followed by reverse transcription and amplification of complementary DNA (cDNA). This step is crucial for scRNA-seq, as it amplifies the limited RNA content of a single cell to a level suitable for sequencing [31]. The amplified cDNA is subjected to high-throughput sequencing platforms such as Smart-Seq and Drop-Seq, each offering distinct advantages depending on the characteristics of the cell population [29, 30, 32, 33]. The resulting sequencing data requires sophisticated bioinformatics processing, including denoising, deduplication, and cell type separation. Dimensionality reduction algorithms like tSNE and computational deconvolution tools are employed to disentangle and quantify cell types within complex tissue samples [34]. Analysis of SCS data provides valuable information about gene expression profiles, functional states, and cellular heterogeneity. This information contributes to our understanding of disease onset and progression in fields such as oncology, immunology, and developmental biology [22, 35]. SCS technology enables comprehensive analysis of individual cells, uncovering cellular complexity and diversity. However, challenges such as complex data processing and high costs necessitate continuous optimization of experimental design and analytical methods (Fig. 1).

### Evolution of single-cell sequencing technology

Initially, life sciences research predominantly relied on the analysis of cell populations or tissue samples. Traditional sequencing methods, such as DNA sequencing and bulk RNA sequencing, were applied to these samples [36–39]. However, these approaches were prone to obscuring biological differences between cells due to sample averaging and technical noise [40]. As technological advancements progressed, the importance of studying individual cells became evident, leading to the development of SCS technologies. The inception of SCS can be traced back to 2001, marking a groundbreaking advancement in the field [41]. Since then, the technology has evolved and become widely adopted. Between 2010 and 2019, there was a notable increase in publications on SCS, driven by the development of high-throughput sequencing platforms and novel computational algorithms [35]. Next-generation sequencing platforms that emerged in 2005 significantly improved methodologies for SCS [42]. In 2009, Tang et al. achieved mammalian cell transcriptome sequencing for the first time, representing a pivotal milestone in scRNA-seq [43]. In 2011, Navin et al. published the first study on the genome sequencing of individual human cells, further propelling the advancement of SCS [44].

The application of SCS in cancer research has been particularly prominent. For instance, since 2016, Tirosh et al. have utilized scRNA-seq to reveal the multicellular ecosystem of metastatic melanoma, demonstrating the immense potential of this technology in cancer immunology research [45]. Additionally, scRNA-seq has been extensively applied to study tumor heterogeneity, immune cell dynamics, and cancer metastasis, providing new tools and perspectives for understanding disease mechanisms and developing personalized treatment strategies [10].

Recent years have witnessed continuous progress in SCS technologies. For example, strategies such as barcoding, multiplex barcoding, and CRISPR barcoding have enabled mitochondrial tracking at the single-cell resolution, methods that have been validated in animal development and stem cell research [46]. Furthermore, SCS has demonstrated its powerful capabilities in revealing cellular heterogeneity and dynamic changes in complex biological systems, with applications in neurodegenerative diseases, autism spectrum disorders, cardiovascular diseases, and more [10, 47].

### Advancements in single-cell sequencing technology

With ongoing technological advancements, SCS has made significant strides in addressing challenges such as complex data processing, high costs, and sequencing accuracy. For instance, droplet-based technologies and high-throughput SCS platforms, including Drop-seq, InDrop, CEL-seq, and the 10X Genomics Chromium system, have been employed to reduce costs [48–51]. The combination of pooled CRISPR with scRNA-seq enables the construction of genetic networks and facilitates large-scale combinatorial functional loss-of-function screens. This approach further reduces costs by minimizing the number of experiments and sample requirements [52–57].

The development and application of more efficient computational methods, such as clustering algorithms, anomaly detection algorithms, self-supervised learning, and deep learning have improved the accuracy and efficiency of data analysis [58–64]. New technologies like microfluidic platforms or microfluidics-free approaches protect cells from shear stress, enhancing cell viability and sequencing quality [65–69]. These advancements not only increase the success rate of experiments but also decrease reliance on costly equipment. Single-cell T cell receptor sequencing (scTCR-seq) and single-cell B cell receptor sequencing (scBCR-seq) enables the sequencing of T cell and B cell receptors at the individual cell level. These methods are utilized to analyze the diversity and clonality of the immune cells, as well as to track their dynamic changes in various diseases and immune responses. By providing insights into the immune cell repertoire, scTCR-seq and scBCR-seq offers powerful tools for immunological research and clinical applications [70–75].

#### Advancements in single-cell multi-omics

In recent years, SCS has made significant strides, expanding its scope beyond gene expression analysis to include the study of epigenetic characteristics at the single-cell level, such as chromatin structure and regulatory elements. Techniques like single-cell chromatin accessibility (scATAC-seq) are now commonly used. And over the past few years, advancements by various research groups have enabled the examination of histone modifications and transcription factor (TF) interactions with DNA at a resolution that captures individual cell details. Notably, the laboratories of Henikoff and Castelo-Branco have employed the 10X Genomics scATAC-seq platform to pioneer a single-cell CUT&Tag technique, which facilitates the dissection of histone mark patterns within solitary cells. The emergence of multimodal chromatin profiling techniques, including nanoCT and Nanobody-tethered transposition (NTT)-seq [76], has advanced the field by enabling the concurrent evaluation of multiple histone modifications within the context of a single cell. Moreover, the integration of single-cell genomics and epigenomics allows for a detailed elucidation of gene regulatory networks and epigenetic variations. These innovative profiling strategies are pivotal for exploring the intricate interplay between chromatin states and cellular dysregulation in various pathologies, opening avenues for the discovery and application of new epigenetic treatments [77–86]. However, sophisticated methodologies for 3D genome profiling at the single-cell level, such as scMicro-C and scNanoHi-C, necessitate expertise and specialized instrumentation for their execution [87]. Additionally, SCS can assess the mitochondrial status of individual cells, with more precise methods being employed to detect mitochondrial function [88–93]. Single-cell metabolomics (scMetabolomics) analysis techniques, including scMetabolomics SCENITH, enable the study of metabolic pathways and products within individual cells, thereby revealing their metabolic state [94–97]. Furthermore, single-cell proteomics (scProteomics) allows for the detection of proteins within each cell among a large population, providing a comprehensive understanding of protein expression changes in both health and disease [98–103]. By leveraging approaches like CITE-seq, which amalgamate cell surface protein quantification with scRNA-seq, our comprehension of cellular protein markers and functional proteins has been significantly expanded, yielding a more holistic depiction of cell states [104]. Building on the need to detect intracellular proteins, a suite of innovative techniques has emerged in the wake of CITE-seq and ECCITE-seq, utilizing antibody-based barcoding to probe for intracellular protein levels. Techniques such as ASAP-seq have adopted droplet technology, initially designed for ATAC-seq, to access intracellular proteins through formaldehyde fixation and NP-40 permeabilization. inCITE-seq has similarly broadened the scope of scRNA-seq to include the analysis of TFs treated with a FA-NT cocktail for simultaneous fixation and permeabilization. Additionally, plate-based assays like RAID have been complemented by droplet-based systems, including QuRIE-seq, to identify phosphorylated proteins, thus quantifying intracellular signaling events. These methodological enhancements have allowed for a more integrated analysis of cell surface and intracellular proteins, providing a deeper insight into cellular dynamics and functionality [105–108]. By amalgamating single-cell genomic, transcriptomic, proteomic, and epigenetic data, a more holistic understanding of cellular heterogeneity and function can be achieved. The synergy of technologies such as scRNA-seq, scDNA-seq, scATAC-seq, scProteomics, and scMetabolomics unveils the intricate relationships between different cell types. This kind of multi-omics approach allows for a more comprehensive grasp of the pathological changes in various cell types during disease, spanning from genetic to chromatin, protein, and metabolic levels [74, 96, 109, 110].

#### Advancements in the integration of spatial omics and single-cell sequencing

While SCS can elucidate the genetic, transcriptional, and proteomic characteristics of cells at the individual level, it lacks spatial context, which may lead to incomplete information and potentially misleading conclusions. The integration of SCS with spatial omics technologies adeptly addresses this limitation, providing a comprehensive view of cellular heterogeneity within its spatial framework. This combined approach allows researchers to discern the spatial relationships and interactions among cells, thereby enhancing the understanding of cellular function and organization in health and disease. Spatial transcriptomics (ST) technology, through high-resolution imaging and analysis, is capable of revealing the molecular dynamics between cells and within tissues, allowing for precise analysis of cellular transcriptional profiles. Moreover, it quantifies and visualizes the heterogeneity of the tumor microenvironment (TME) [111, 112]. For instance, tools like spatialGE leverage spatial transcriptomics to quantify and visualize the heterogeneity within the TME, highlighting gene expression differences at both intercellular and intracellular levels [113, 114]. This technology offers new perspectives for understanding the origins, progression, and treatment of tumors. Additionally, ST can elucidate cell–cell interactions between tumor cells and their immediate microenvironment. For example, by integrating ST, scRNA-seq, or single-nucleus RNA (snRNA-seq) sequencing, it is possible to map the transcriptomic and cellular architecture of tumors, thereby understanding the cell–cell interactions between tumors and their immediate microenvironment [115–120].

## Single-cell sequencing technology advances renal cancer research

### Exploring the origins of renal cancer epithelial cells with single-cell sequencing technology

#### Clear cell renal cell carcinoma

Exploring the origins of tumor cells is a crucial step in oncology research, as it provides insights into the development of cancer. Recent studies using single-cell transcriptomic analysis have shed light on the possible origin of ccRCC from proximal tubule (PT) cells within the kidney [121]. Transcriptomic similarities between ccRCC cells and PT cells suggest a potential lineage relationship [122]. Spatial RNA sequencing techniques, such as Decoder-seq, have further demonstrated that the tumor region of ccRCC is enriched with PT cells [123]. Furthermore, analysis of cell–cell communication has revealed that a subpopulation of PT cells in ccRCC plays a central role in intercellular signaling interactions with endothelial ascending vasa recta cells, vascular smooth muscle cells (vSMCs), other PT cells, and intercalated cells (ICs). The epidermal growth factor (EGF)-epidermal growth factor receptor (EGFR) pathway has been implicated in these interactions [124]. These findings further support the hypothesis that ccRCC originates from PT cells [125–129]. Notably, VCAM1 + PT cells, which are exclusive to normal tissue, bear the closest resemblance to ccRCC cells, suggesting their involvement in the transition from normal to tumor cells [130]. Advanced analysis utilizing scRNA-seq and spatial genomics has further indicated that the epithelial cell subgroups within ccRCC are analogous to the proximal straight tubules (PST), further supporting the PT as the origin of ccRCC [131]. However, scRNA-seq studies of ccRCC patient tumors have also identified cancer stem cells (CSCs) located at the divergence point of various tumor cell subpopulations. These CSCs exhibit gene expression profiles highly similar to the bulk tumor cells, suggesting their potential role in the origin and maintenance of ccRCC [132].

These studies highlight the complexity of ccRCC origins, with evidence supporting both PT cells and CSCs as potential cellular sources. Further investigation is needed to fully elucidate the precise mechanisms underlying ccRCC development and the contributions of different cell populations in tumor initiation and progression.

#### Papillary renal cell carcinoma

Single-cell transcriptomic analysis of renal tumors has provided insights into the origin of type 1 papillary renal cell carcinoma (pRCC) cells. Similar to ccRCC, pRCC cells have been found to share similarities with PT cells, suggesting a potential common origin for both pRCC and ccRCC from PT cells [121]. Human renal progenitor cells consist of multipotent progenitors (CD133 + VCAM-1 +, located at the urinary pole of the renal vesicle) and committed renal tubular progenitors (CD133 + VCAM-1-). scRNA-seq analysis has revealed that PT cells in pRCC exhibit characteristics similar to human renal progenitor cells, supporting the notion that pRCC cells may originate from these progenitor cells [133]. While most evidence points towards the PT as the source of pRCC cells [121, 134, 135], heterogeneity in the origins of pRCC cells has also been observed through the use of scATAC-seq. In addition to deriving from the classical proximal renal tubules, pRCC cells may also arise from the principal cells of the collecting ducts [136].

#### Chromophobe renal cell carcinoma

Chromophobe renal cell carcinoma (chrRCC) is the second most common subtype of non-clear cell renal cell carcinoma (nccRCC), and it is believed to have a distinct cellular origin compared to ccRCC [137]. Decoder-seq analysis has revealed that the tumor region of chrRCC is enriched with distal tubule epithelial cells [123]. Similarly, scRNA-seq analysis indicates that chrRCC shows the highest similarity to ICs, suggesting an origin in the distal renal unit [122, 134]. Intercellular communication studies have highlighted a particular subset of ICs within chrRCC that play a crucial role in initiating signaling interactions. These ICs engage in intricate communication networks with various cell types, including endothelial cells (ECs) of the ascending vasa recta, vascular smooth muscle cells (vSMCs), other ICs, and PT cells. Notably, the EGF-EGFR signaling pathway, which has been implicated in clinical research, supports the theory that chrRCC originates from ICs [124]. These findings provide further evidence supporting the hypothesis that chrRCC originates from the distal nephron units [138].

#### Wilms tumor

Analyses of scRNA-seq data from Wilms tumors have uncovered a resemblance between these malignant cells (MCs) and their normal fetal counterparts, such as the cells of the ureteric bud and nephrogenic vesicles, which are part of the developing kidney. The presence of distinct Wilms tumor cell populations that mirror these fetal cell types suggests an evolutionary lineage from healthy fetal cells [121], which is consistent with the findings of Stupar et al., which indicate that nephroblastoma may also originate from the ureteric bud [139]. Peired et al. also discovered a high degree of similarity between Wilms tumors and early renal units, indicating their association with the development of early renal units [133]. These discoveries are in accordance with the research conducted by Aiden et al., which suggests that Wilms tumor may originate from normal renal stem cells [140]. The application of scRNA-seq has enabled the classification of Wilms tumor cells into multiple distinct subgroups. It has been observed that cells positive for both sine oculis homeobox 2 (SIX2) and Cbp/p300-interacting transactivator with Glu/Asp-rich carboxy-terminal domain 1 (CITED1) tend to be associated with earlier developmental stages and exhibit characteristics of CSCs. This suggests that SIX2 + CITED1 + cells may serve as the cells of origin for nephroblastoma [141].

#### Other rare subtypes of renal cell carcinoma

Through multi-omics analysis, it has been identified that translocation renal cell carcinoma (TRCC) and eosinophilic solid and cystic renal cell carcinoma (ESCRCC) most likely originate from PT cells, while renal oncocytoma (RO) shows the highest similarity to ICs, suggesting an origin from the distal nephron. In renal angiomyolipoma (AML), tumor cell subpopulations resemble vSMCs and ECs [134]. In single-transcriptome analyses of hereditary renal cancers, hereditary leiomyomatosis and renal cell carcinoma (HLRCC) are likely to originate from PT cells, while Birt-Hogg-Dubé-associated kidney cancer (BHD-RCC) likely originates from collecting duct cells, consistent with the research by Jikuya et al., which suggests that BHD-RCC and sporadic chrRCC originate from distinct cell types [142]. Furthermore, von Hippel-Lindau-associated kidney cancer (VHL-RCC) and sporadic clear cell renal cell carcinoma (s-RCC) cells cluster with glomerular/vascular cells, suggesting a glomerular/vascular origin for VHL-RCC and s-RCC [143]. In renal medullary carcinoma (RMC), scRNA-seq analysis conducted by Vokshi et al. revealed a high degree of similarity between RMC cells and cells of the thick ascending limb (TAL), indicating a likely origin from TAL cells [144]. For collecting duct renal cell carcinoma (CDRCC), CSCs possess multilineage differentiation capabilities and self-renewal abilities. They play a central role in the differentiation process, being able to transform into both primary CDRCC cells and metastatic cells, suggesting that CDRCC cells may originate from CSCs. However, further investigation is needed to determine the precise identity of the original CSCs [145].Li et al. conducted single-cell exon sequencing on a renal cancer patient's tumor and found that CD133 + RCC cells exhibit distinct cancer stem cell (CSC) characteristics, suggesting that they are more likely to originate from cancer cells than from normal cells. This finding provides evidence for the origin of CSCs [146].

### Single-cell sequencing technology contributes to a deeper understanding of the mechanisms underlying the initiation and progression of renal cancer

#### Single-cell sequencing technology reveals renal cancer epithelial-mesenchymal transition and metastasis dynamics

The disruption of the epithelial-mesenchymal transition (EMT) process plays a significant role in cancer progression, enhancing the invasive and metastatic capabilities of cancer cells [147, 148]. scRNA-seq analysis across various cancers, including ccRCC, has identified meta-programs (MPs) reflecting coordinated gene expression changes among cells and contributing to tumor transcriptional heterogeneity. Notably, the presence of mesenchymal-like and EMT-associated MPs in ccRCC suggests that EMT is a crucial mechanism in ccRCC progression [149]. Similarly, scRNA-seq, spatial genomics, and non-negative matrix factorization (NMF) have allowed the classification of ccRCC cells into six MPs. MP3, in particular, is enriched with EMT-associated genes such as Transforming growth factor beta-inducible protein (TGFBI) and Metallothionein-2A (MT2A). Cells with PT features show an inverse correlation with high expression of EMT genes. Importantly, EMT-high tumor cells are more prevalent at the tumor-normal tissue interface and tumor margins, indicating a more aggressive and migratory phenotype in ccRCC cells [150]. ST analysis of RCC tissues using the Decoder-seq technique has classified RCC cells into seven cellular niches (CNs), with CN5 showing upregulated EMT genes. This gradient of EMT gene expression expands outward from the tumor core (T) to the invasive margin (IM) and then to the normal region (SN). A shift in transcriptomic activity is observed, with higher activity in the IM and SN areas compared to the T area, suggesting a greater proliferative capacity of cells at the tumor margin [123]. Furthermore, analysis of single-cell data from ccRCC using the cancer-finder tool identified ten significant malignancy-associated genes enriched at the tumor-normal tissue interface. These genes, including immunoglobulin-related genes, Transgelin (*TAGLN*), Keratin 19 (*KRT19*), Metastasis-associated lung adenocarcinoma transcript 1 (*MALAT1*), and superoxide dismutase (*SOD2*), play a role in the EMT process in various cancers. Their expression patterns suggest that EMT-associated programs are enriched at the periphery of RCC, facilitating invasion into adjacent normal tissues [151]. These findings suggest that EMT-associated programs are more enriched at the periphery of RCC, thereby mediating the invasion of renal cancer into adjacent normal tissues. Additional research indicates that EMT-associated cancer cells not only localize to the tumor-normal tissue interface but also coexist with surrounding endothelial and fibroblast cells. This suggests that interactions between cancer cells and stromal cells at the tumor margin contribute to the invasion and metastasis of ccRCC [152]. Analysis of scRNA-seq data from a range of cancers, including RCC, has identified three distinct EMT-associated recurrent heterogeneity programs (RHPs). EMT-II is characterized by genes associated with cellular motility and partial EMT phenotypes, such as fibronectin (*FN1*), vimentin (*VIM*), and AXL receptor tyrosine kinase. EMT-III is defined by genes related to cell adhesion, including *laminins A3*, *C3*, and *B3*, and plakoglobin (*JUP*), which has been implicated in facilitating the collective invasion of aggressive metastatic circulating tumor cells [153]. These findings suggest the involvement of EMT-II and EMT-III in the EMT process of RCC. Employing one-class logistic regression (OCLR) algorithm, Malta et al. established mRNA-based (mRNAsi) and DNA methylation-based (mDNAsi) stemness metrics from single-cell data. Notably, mRNAsi levels were significantly higher in metastatic RCC samples compared to primary tumors. Metastases in the prostate and pancreas showed enhanced dedifferentiation, aggressiveness, and resistance to therapy, indicating alterations in stemness during RCC metastasis and its potential contribution to metastatic potential [154]. ScRNA-seq and spatial transcriptomics have revealed a positive correlation between mesenchymal-like ccRCC cells (ccRCC.mes) and myofibroblast-like cancer-associated fibroblasts (myCAFs), serving as predictive markers for poor patient prognosis. These cells are located near the tumor-normal tissue interface and are associated with multiple signaling pathways, suggesting their role in promoting ccRCC invasion and metastasis [131]. ScRNA-seq analysis of RCC tumor cells has uncovered two metastasis-related gene expression programs. The Metastasis-I program, characterized by extracellular matrix (ECM)-related genes and EMT features, is observed across multiple malignant cell subgroups and is associated with enhanced invasiveness and migration. In contrast, the Metastasis-II program, which includes some ECM genes, lacks the expression of key EMT TFs like snail family transcriptional repressor (SNAIL), snail family transcriptional repressor 2 (SLUG), and twist basic helix-loop-helix transcription factor (TWIST), and shows a subtle shift in epithelial gene expression without a global change in epithelial markers, suggesting a distinct, non-canonical EMT process that may play a unique role in tumor metastasis [155]. Integrating single-cell transcriptomic data from ccRCC revealed a significant enrichment of metastasis-associated genes in T cells and macrophages. Further analysis indicated that pro-inflammatory (M1-like) macrophage subsets and exhausted T cells may promote ccRCC metastasis [156], contradicting the conventional view that pro-inflammatory (M1-like) macrophages are associated with anti-tumor effect [157–161]. This highlights the complex role of pro-inflammatory (M1-like) macrophages in ccRCC metastasis. In bone metastatic RCC (BMRCC), analysis of single-cell and bulk transcriptomic data has shown high expression and significant correlation of epithelial membrane protein 1 (EMP1) and collagen type III alpha 1 chain (COL3A1) in fibroblasts. EMP1 +/COL3A1 + fibroblasts are implicated in the bone metastasis of renal cancer and are associated with the activation of EMT genes and the wingless-type MMTV integration site family (WNT) signaling pathway. BMRCC is also characterized by altered differentiation trajectories of myeloid cells, particularly macrophages, exhibiting M2 polarization and pro-angiogenic characteristics. Interactions, such as those mediated by signal regulatory protein alpha chain (SIRPα)- integrin-associated protein (CD47), between macrophages and immunosuppressive T cells influence tumor progression [162, 163]. Intriguingly, EMP1 + CAFs are also linked to breast cancer metastasis [164].

The remodeling of the tumor ECM is closely associated with cancer invasion, metastasis, poor prognosis, and angiogenesis [165–174]. scRNA-seq reveals that stromal cells, including ECs, pericytes, and fibroblasts, undergo changes in ccRCC that compromise vascular integrity and contribute to vascular remodeling. The EMT and angiogenesis signature scores of these stromal cells differ significantly from those in normal renal tissue, suggesting a relationship between altered stromal cells, vascular remodeling, and EMT in ccRCC [175]. Analysis of primary tumors (PT) and tumor thrombus (TT) in RCC using scRNA-seq has shown extensive interactions between various cell types in the TT, such as macrophages, fibroblasts, ECs, with malignant cells (MCs). These interactions include promotion of ECM remodeling, angiogenesis, and the EMT process [176]. Additionally, tip cell-like ECs have been identified in ccRCC and are involved in ECM remodeling and suppression of immune cell function within the TME. These tip cell-like ECs highly express genes associated with EMT, suggesting their role in promoting the EMT process [177].

In nccRCC, scRNA-seq has provided insights into the underlying mechanisms. In RMC, RMC cells exhibit a gradient of EMT characteristics, encompassing both epithelial-like and mesenchymal-like cell subpopulations with distinct gene expression profiles and functions. Furthermore, the loss of transcription factor CP2-like 1 (TFCP2L1) and the activation of myelocytomatosis oncogene (*MYC*) lead to a series of gene expression alterations, including the downregulation of epithelial markers and the upregulation of mesenchymal markers, thereby promoting the EMT process [144]. In CDRCC, scRNA-seq analysis of bone metastases has revealed complex interactions between MCs and bone-related cells. MCs promote the proliferation and differentiation of monocytes into osteoclasts, inhibit the proliferation of mesenchymal stem cells in the bone marrow, and facilitate bone destruction. Additionally, CSCs play a significant role in regulating the bone microenvironment and the osteotrophic spread of CDRCC [145].

#### Single-cell sequencing technology deciphers the metabolic reprogramming in renal cancer

Metabolic reprogramming is a characteristic feature of renal cancer [178–182], particularly in ccRCC, which has been recognized as a metabolic disease [183–185]. Integrating scRNA-seq with multi-omics has led to the discovery of a distinct ccRCC variant known as de-clear cell differentiation (DCCD)-ccRCC. Unlike typical ccRCC, which is marked by lipid accumulation [186–189], DCCD-ccRCC exhibits reduced lipid levels due to diminished β-oxidation. Genes involved in fatty acid, amino acid, and carbohydrate metabolism are downregulated, indicating metabolic reprogramming in this subtype. Activation of Glutathione (GSH) metabolism in DCCD-ccRCC leads to increased GSH and glutathione disulfide (GSSG) levels, neutralizing reactive oxygen species (ROS) and promoting cell proliferation and therapy resistance. Upregulation of nutrient transporter genes allows heightened nutrient uptake for cell growth, highlighting the role of metabolic reprogramming in ccRCC progression [190]. Pan et al. categorized ccRCC cells into five subgroups along distinct differentiation trajectories using scRNA-seq. Aberrant lipid metabolism and accumulation are observed in ccRCC, but metabolic heterogeneity exists among the subgroups. The ccRCC5 subgroup exhibits accelerated lipid accumulation through biosynthesis, storage, and transport, while ccRCC2 and ccRCC3 subgroups upregulate fatty acid oxidation (FAO)-related pathways, with FAO having an inhibitory effect on tumor progression [132]. This suggests that metabolic reprogramming in ccRCC is not a homogeneous state but rather a composite of specific metabolic patterns within different tumor subgroups, together constituting the metabolic profile of ccRCC. Additionally, scRNA-seq and spatial transcriptomics in ccRCC have shown that as the disease progresses, copper (Cu) levels significantly increase in blood and tumor tissues. Copper preferentially accumulates in cytochrome c oxidase (CuCOX) within tumor cells, driving complex metabolic reprogramming processes. Cu accumulation increases mitochondrial oxygen consumption rates, activates the pentose phosphate pathway and nucleotide biosynthesis, and induces tight coupling between glucose oxidation and glutathione biosynthesis. This enables tumor cells to manage Cu-related oxidative stress and maintain redox balance, providing a survival and proliferation advantage in a Cu-rich microenvironment [191]. Pan-cancer analyses based on scRNA-seq, including renal cancer, have identified upregulation of glycolysis/gluconeogenesis, propanoate metabolism, steroid hormone biosynthesis, tricarboxylic acid (TCA) cycle, and glucagon signaling pathways across most cancers, while the biosynthesis of glycosaminoglycans is downregulated [192]. During the onset and progression of ccRCC, progenitor PST cells retain characteristics of gluconeogenesis and oxidative phosphorylation (OXPHOS) partially in ccRCC epithelial cells (ccRCC.epi) but diminish during the EMT process. In contrast, hypoxia and glycolytic features are enhanced in MCs, indicating a metabolic reprogramming shift in ccRCC evolution [131]. Similarly, scRNA-seq indicates that the expression of gluconeogenic genes is downregulated in ccRCC and associated with poor prognosis. Restoring gluconeogenesis can inhibit MC proliferation, highlighting the role of inhibiting gluconeogenesis as a key metabolic characteristic in ccRCC development [193]. And it is intriguing to note that the downregulation of the gluconeogenic pathway is also observed in chrRCC [194]. Additionally, there is an enrichment of pathways associated with lipid synthesis and distribution in ccRCC, alongside a suppression of lipid catabolism, aligning with the observed abnormal lipid accumulation in ccRCC tissues and cell lines. Moreover, the breakdown of amino acids and carbohydrates, as well as the peroxisome proliferator-activated receptors (PPAR) signaling pathway, are dampened. Notably, lipid metabolism anomalies are also present in cancer-associated fibroblasts (CAFs), indicating that the metabolic reprogramming in ccRCC affects the TME as a whole, not just the cancer cells [195]. And research on diverse cancers have also demonstrated that metabolic reprogramming is not exclusive to MCs [196–198]. Myofibroblasts and endothelial progenitor cells (EPCs) in ccRCC show enrichment in oxidative respiration pathways, including aerobic respiration and OXPHOS, suggesting their involvement in metabolic reprogramming [199]. Tumor progression is linked to oxidative stress, with increased oxidative processes in MCs and the generation of ROS through OXPHOS posing a threat to cellular DNA and elevating cancer risk [200–205]. ROS may contribute to tumor advancement by influencing various factors, including angiogenesis [196, 206] and immune microenvironment functions [199]. Metabolic reprogramming in ccRCC is not limited to tumor and stromal cells but also occurs in immune cells. scRNA-seq analysis has revealed metabolic reprogramming in T and Nature Killer (NK) cells within the tumor immune microenvironment (TIME). The most notable metabolic alterations were observed in CD4 + and CD8 + T cells, which display distinct metabolic pathway activities. Macrophages in the M2-polarized subset exhibit elevated glycolytic activity, while those in the M1-polarized subset show increased arachidonic acid and glutathione metabolism, as well as fatty acid synthesis. Macrophages with upregulated OXPHOS and tryptophan metabolism are prevalent in HG tumors and associated with functional modifications. Sphingolipid metabolism plays a role in regulating macrophage function, particularly M2 polarization. ST analysis indicates that the activity of metabolic processes such as the citrate cycle, glycolysis, fatty acid metabolism, and tyrosine metabolism is inversely related to the distance from the tumor center. This suggests higher metabolic activity in the tumor core and reduced activity at the periphery. Purine metabolism activity reaches its peak in the intermediate zone between the tumor center and edge, correlating with cell proliferation. AMP, CDP, and fatty acids tend to accumulate at the tumor center; acetyl-CoA, ornithine, and tyrosine are concentrated near the tumor edge; and succinate, glutamate, and fumarate are enriched within the tumor region but exhibit a discontinuous distribution [207]. Furthermore, metabolic alterations in RCC shape the TIME conducive to its progression. Increased degradation of tryptophan, leading to the production of immunosuppressive kynurenine, is a characteristic of metabolic reprogramming in RCC. This can inhibit T cell function and activity, facilitating immune evasion by tumor cells [208]. In various cancers, including renal cancer, immune cells within the TIME exhibit relatively low metabolic activity, which may be associated with cancer progression and immune evasion [192]. Single-cell analysis of ccRCC has shown elevated levels of Nicotinamide-N-methyltransferase (NNMT) in primary ccRCC tissues and metastatic lesions compared to non-cancerous tissues. NNMT influences mitochondrial function and cellular vitality by facilitating glutamine's entry into the tricarboxylic acid (TCA) cycle for mitochondrial OXPHOS Moreover, the use of glutamine for GSH synthesis and ROS scavenging could be a reason for the heightened glutamine requirement observed in ccRCC [209], which further enriches the theoretical framework regarding the glutamine addiction observed in renal cancer [210].

Metabolic reprogramming also plays a crucial role in the progression of nccRCC [180, 211, 212] and exhibits metabolic heterogeneity across different subtypes. Type 1 pRCC tumors downregulate pathways of de novo purine nucleotide synthesis and the TCA cycle, while these pathways are enriched in RO, with an accumulation of glycolytic product pyruvate in RO. The pentose phosphate pathway and heparan sulfate degradation may be upregulated in type 1 pRCC, and fatty acid β-oxidation-related pathways are enhanced in renal AML. Additionally, comparing the metabolomes of high and low weighted gene instability index (wGII) nccRCC samples revealed significant upregulation of six compounds and downregulation of five in the high wGII group. High expression of Pyrroline-5-Carboxylate Reductase-1 (PYCR1), along with elevated levels of proline and NADH, suggests increased proline biosynthesis in high wGII tumors, potentially supporting cancer cell proliferation and survival under hypoxic conditions. In contrast, genomically stable samples express higher levels of glucosamine, glucaric acid, and 8-hydroxyquinoline, derivatives of which possess anti-cancer properties [134]. In CDRCC, glycolysis is upregulated, whereas the CD acid secretion pathway is dampened. In chrRCC, the proteoglycan pathway shows enrichment in cancer [213].

#### Single-cell sequencing technology dissects the immune evasion mechanisms in renal cancer

Tumor cells evade immune surveillance through various mechanisms, which is a significant factor in cancer progression [214–221], and renal cancer is no exception [210, 222–224], exhibiting phenomena of immune evasion that contribute to disease progression and resistance to immunotherapy.

Detailed research on the TIME of ccRCC using scRNA-seq and scTCR-seq has revealed progressive exhaustion of CD8 + T cells and M2 polarization of macrophages during disease progression. There is an interaction between M2-like macrophages and exhausted CD8 + T cells. M2-like macrophages express multiple ligands, including poliovirus receptor (PVR), CD80, CD86, Galectin-9 (LGALS9), Programmed cell death-ligand 1 (PD-L1), and nectin cell adhesion molecule 2 (NECTIN2), associated with T cell inhibition. Exhausted CD8 + T cells express factors that promote M2-like polarization, such as macrophage migration inhibitory factor (MIF) and colony-stimulating factor-1 (CSF1). This creates a positive feedback interaction loop leading to sustained mutual suppression of macrophage and T cell functions within the TIME, potentially causing immune evasion in ccRCC [225]. In the context of immune checkpoint blockade (ICB) therapy, ccRCC cells upregulate nectin-2 along with T cell checkpoint molecules and molecules involved in suppressing macrophage inflammation and evading phagocytosis. Specifically, MCs interact with CD8 + T cells and tumor-associated macrophages (TAMs) through the engagement of LGALS9 with T cell immunoglobulin and mucin domain 3** (**TIM-3), thereby inhibiting the cytotoxic activity of CD8 + T cells and inducing the secretion of growth factors by TAMs. MCs also express nectin-2, which binds to the co-inhibitory receptor T-cell immunoglobulin and immunoreceptor tyrosine-based inhibitory motif domain (TIGIT) on CD8 + T cells. Additionally, MCs suppress T cell activation by expressing secreted phosphoprotein 1 (SPP1) and interacting with CD44 on CD8 + T cells. They promote the secretion of growth factors by TAMs and inhibit phagocytosis by expressing MIF and CD47, which interact with CD74 and SIRPα on TAMs, respectively [226]. These interactions mediated by malignant cells contribute to resistance to ICB therapy and immune evasion by suppressing the immune functions of TAMs and T cells. Analysis of ccRCC through scRNA-seq has identified a macrophage subset that is closely associated with disease recurrence, characterized by the upregulation of proteins such as triggering receptor expressed on myeloid cells 2 (TREM2), apolipoprotein E (APOE), and Complement Component 1, Q subcomponent (C1Q) [227]. It has been well-documented that C1Q + macrophages associated with tumor advancement and unfavorable outcomes across a spectrum of cancers [228–231]. Notably, study has suggested that TREM2 + macrophages may promote tumor progression by inhibiting T cell proliferation in other cancer [232], implying that TREM2 + macrophages in ccRCC may also contribute to tumor immune evasion through this mechanism, thereby becoming one of the factors in disease recurrence. These discoveries corroborate the view put forth by other researchers that TREM2 + macrophages contribute to the promotion of tumor progression [233–236].

**Fig. 2:**
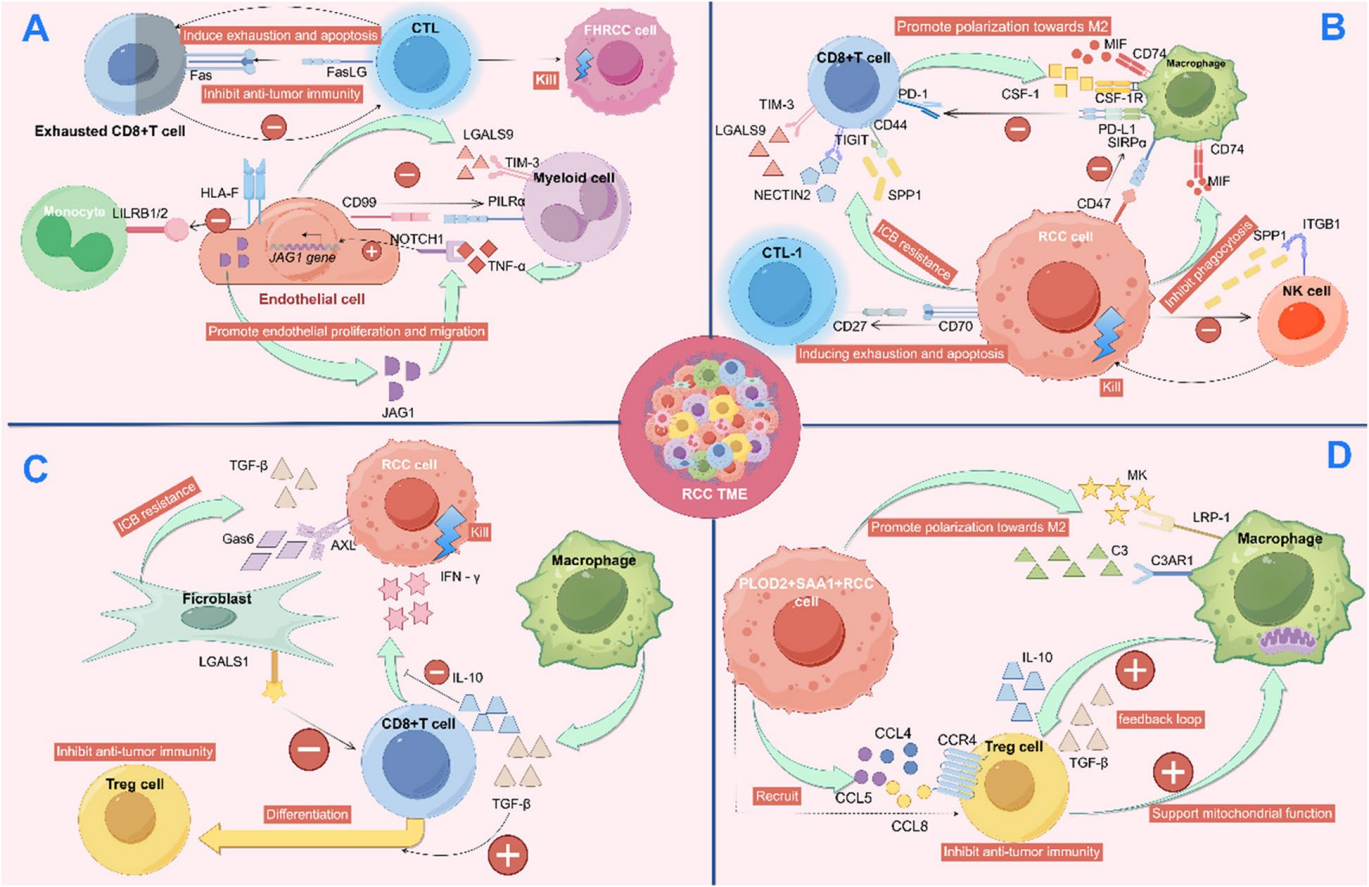
The immune evasion mechanisms in renal cancer revealed by single-cell sequencing. **A** In FHRCC, the interaction between exhausted CD8+ T cells and CTL facilitates the exhaustion and apoptosis of CD8+ T cells through the Fas/Fas ligand pathway, thereby inhibiting their anti-tumor immune response. In ccRCC, endothelial cells engage in complex intercellular communications with myeloid cells. Specifically, endothelial cells interact with myeloid cells through CD99 binding to the paired immunoglobulin-like 2-type receptor α (PILRα), leading to a reduction in immune cell infiltration levels. Furthermore, endothelial cells exert an immunosuppressive effect on myeloid cells via the LGALS9-TIM-3 axis, while myeloid cells promote the expression of Jagged (JAG) 1 on endothelial cells through the secretion of TNF-α acting on NOTCH1 receptors. JAG1, in turn, enhances the NOTCH signaling pathway, thereby promoting the proliferation and migration of endothelial cells within ccRCC. Endothelial cells also express HLA-F, which acts on leukocyte immunoglobulin-like receptor B (LILRB) 1/2 on monocytes, suppressing their immune functions. These sophisticated interactions between endothelial and myeloid cells ultimately contribute to immune evasion in ccRCC. **B** In ccRCC, tumor cells suppress immune cells, leading to immune evasion. Tumor cells employ various mechanisms to inhibit NK cells, induce exhaustion and apoptosis in CD8+ T cells, mediate resistance to ICB therapy, and suppress the function of macrophages. Moreover, interactions between exhausted CD8+ T cells and macrophages result in further suppression of CD8+ T cells and polarization of macrophages towards the M2 phenotype. **C** Macrophages induce the conversion of T cells into Tregs, and both macrophages and fibroblasts suppress the immune response of T cells against RCC tumor cells. Additionally, fibroblasts mediate the resistance of tumor cells to ICB therapy. This complex interplay between macrophages, T cells, and fibroblasts contributes to immune evasion in the TME. **D** PLOD2 + SAA1 + RCC cells promote the polarization of macrophages towards the M2 phenotype through multiple signaling pathways, potentially recruiting Tregs to induce immune suppression. Tregs support mitochondrial function in macrophages, while macrophages, in turn, enhance Treg function, forming a feedback loop that mediates immune evasion. This figure was created based on the tools provided by Figuredraw.com

scRNA-seq analysis suggests that MCs modulate immune functions by highly expressing SPP1, which acts on the receptor integrin β1 (ITGB1) of NK cells and integrin α4 (ITGA4) of cytotoxic T lymphocyte (CTL)−1 cells. MCs express high levels of CD70, which interacts with the receptor CD27 on CTL-1 cells, inducing T cell exhaustion and apoptosis, potentially facilitating immune evasion [175]. Regulatory T cells (Tregs) are instrumental in the immune evasion strategies employed by tumors [237–239]. Analysis of the TIME in renal cancer through scRNA-seq has revealed a close interaction between Tregs and macrophages. Macrophages may indirectly suppress the function of effector T cells by inducing the proliferation and enhancing the activity of Tregs. In turn, Tregs support the mitochondrial function of macrophages and promote their proliferation, creating a feedback loop that fosters an immunosuppressive environment conducive to tumor survival [240]. Liu et al., through scRNA-seq, identified a subset of MCs in ccRCC characterized by high expression of procollagen-lysine 2-oxoglutarate 5-dioxygenase 2 (PLOD2) and serum amyloid A 1(SAA1), and significantly involved in initiating signaling pathways for SPP1, MIF, pleiotrophin (PTN), and midkine (MK). These MCs interact with TAMs through ligand-receptor pairs such as MIF-CD74, C-X-C motif chemokine receptor 4 (CXCR4)-complement component 3 (C3), and MK-low-density lipoprotein receptor-related protein (LRP)1. Patients with advanced TNM stage exhibit an enrichment of these malignant cells and increased expression levels of C3, positively correlating with the infiltration of M2-like TAMs and Tregs. These cell–cell communications may promote M2 polarization of TAMs, recruit Tregs, and establish an immunosuppressive environment conducive to immune evasion [241]. This suggests that these cell–cell communications may promote M2 polarization of TAMs, recruit Tregs, and establish an immunosuppressive environment conducive to immune evasion. Stromal cells also contribute to the immunosuppressive microenvironment. ECs interact with myeloid cells through CD99–paired immunoglobulin-like type 2 receptor α(PILRα), leading to reduced levels of immune cell infiltration. Additionally, ECs exert immunosuppressive effects on myeloid cells via LGALS9 – TIM-3 [130], thereby playing a significant role in immune evasion. In fumarate hydratase-deficient renal cell carcinoma (FHRCC), immune evasion is prominent. Under ICB therapy, interactions between exhausted CD8 + T cells and CTL dominate cellular communication. Patients with progressive disease (PD) exhibit stronger interactions between CD8 + T cells and CTL cells through the Fas/Fas ligand (FasLG) pathway, inducing exhaustion and apoptosis in CD8 + T cells. The intensity of GALECTIN pathway interactions is heightened, with LGALS9 serving as an immunosuppressive signal [242]. This suggests that the interactions among CD8 + T cells in FHRCC may be a key mechanism underlying resistance to immunotherapy and immune evasion. These mechanisms of the immune evasion are summarized in Fig. 2.

#### Single-cell sequencing technology depicts the landscape of gene expression regulation in renal cancer

Elucidating the epigenetic regulation landscape is crucial for understanding the biology of tumors, uncovering their mechanisms of development and progression, and identifying potential therapeutic strategies in cancer research [243–249]. snATAC-seq analysis across 11 cancer types, including ccRCC, reveals that accessible chromatin regions (ACRs) are mainly in intergenic (49%), promoter regions (8.6%), and distal intergenic (30.8%). In ccRCC cells, chromatin accessibility changes occur near genes like solute carrier family 38 member 8 (*SLC38A8*), musculoaponeurotic fibrosarcoma oncogene homolog A (*MAFA*), and class III β-tubulin (*TUBB3*), with increased accessibility noted. The enhancer region of vascular endothelial growth factor A (*VEGFA)* also shows increased accessibility, while genes like egl-9 family hypoxia-inducible factor 3 (*EGLN3*) exhibit cancer-type-specific changes in chromatin accessibility, correlating with ccRCC pathological features and suggesting a role in its development. During ccRCC metastasis, TFs such as gata—binding protein 6 (GATA6) show altered activity, potentially aiding migration and invasion of ccRCC cells. *TP53* mutations may affect chromatin accessibility near binding sites, influencing cell behavior, such as with gene growth differentiation factor 15 (*GDF15)*. Telomerase reverse transcriptase (TERT) promoter hotspot mutations (*C228T* and *C250T*) show distinct accessibility patterns in ccRCC compared to normal cells, correlating with *TERT* gene expression, indicating an interplay between epigenetic and genetic factors in ccRCC [250]. Della Chiara et al. examined the chromatin accessibility of acquired colorectal cancer (CRC) enhancers associated with Yes-associated protein (YAP) and transcriptional co-activator with PDZ-binding motif (TAZ) across various cancer types, including ccRCC and pRCC. They identified 46 enhancer regions (23%) that were consistently accessible in all cancer types, suggesting a pan-cancer enhancer core. Changes in chromatin accessibility correlate with gene transcription regulation, indicating shared transcriptional mechanisms across different cancers. These conserved regions may serve as binding sites for TFs like YAP/TAZ, affecting gene expression and tumor cell behavior common to multiple cancers [251]. This highlights the role of these pan-cancer enhancer regions with high accessibility in renal cancer development and progression. Single-cell exon sequencing of ccRCC has revealed that the desmoyokin (*AHNAK)* gene plays a significant role in the development of ccRCC. AHNAK is a protein subjected to lysine acetylation, which plays a role in chromatin remodeling [252, 253]. An interaction between the AHNAK protein and the hypoxia-inducible factor 1-alpha (*HIF1*α) gene, crucial for angiogenesis in ccRCC, has been observed. Mutations in the *AHNAK* gene may alter pathways related to lysine acetylation/histone deacetylation or chromatin remodeling, thus affecting the expression regulation of the EGFR pathway and leading to malignant proliferation of renal cells [254]. Furthermore, scATAC-seq has identified cell subtype-specific chromatin accessibility regions in ccRCC, with increased chromatin openness at protein-coding genes specific to tumor cells[such as heparanase 2 (*HPSE2)*, attractin—like 1 (*ATRNL1)*, immunoglobulin superfamily member 21 (*IGSF21)*], rRNA, and certain long non-coding RNAs (lncRNAs) (such as RP11-661C8.2, CTB-164N12.1) [255]. This suggests that these proteins and RNAs may play a role in the development and progression of ccRCC. Saout et al. constructed a tumor clonal lineage through scRNA-seq of ccRCC and classified malignant cells into five distinct subgroups. They observed an evolutionary trajectory of ccRCC cells, with increasing burden of copy number variations (CNVs) indicating accumulating chromatin alterations within cells. In patients with the highest CNV burden, a significant number of differentially expressed genes were found between MCs and ECs, and the tumor lineage accumulated characteristics associated with invasiveness, particularly in subgroup c8. This suggests that chromatin alterations in ccRCC MCs represent an accumulating process from indolent to aggressive disease, potentially influencing gene expression to acquire molecular traits for tumor progression [256]. Multi-region single-cell proteogenomic research has discovered that chemokines such as C-X-C motif chemokine (CXCL)10 and CXCL11 are predominantly expressed by immune cells, while chemokines like C–C motif chemokine ligand (CCL)4 and CCL5 are expressed by malignant cells. The expression of these cytokines is negatively correlated with CpG methylation in their respective promoter regions. In tumors enriched with immune cells, the methylation levels of CCL4 and CCL5 promoters are the lowest, and higher expression levels of CCL4 and CCL5 may attract more immune cell infiltration and enhance immune responses, correlating with good responses to immunotherapy in these patients. Methylation plays a significant role in ccRCC by influencing cytokine expression and cell–cell interactions, impacting the composition and spatial architecture of the TME, and being closely associated with tumor evolution and patient treatment responses [257]. In ccRCC, the *VHL* is often inactivated due to mutations [222, 258–266], and the ras association domain family member 1 A (*RASSF1A*) gene is frequently silenced by chromosomal 3p deletions and promoter hypermethylation [267–272]. Catalano et al. investigated the effects of *VHL* and *RASSF1A* deficiency using scRNA-seq and found that while double knockout of *VHL* and *RASSF1A* was not sufficient to induce ccRCC, it did lead to DNA damage, chromosomal instability, and alterations in gene expression. The absence of *RASSF1A* induced the expression of genes associated with ccRCC, suggesting that its inactivation in human ccRCC may be linked to changes in gene expression patterns [273]. The development of databases based on single-cell epigenetic data has greatly facilitated researchers in exploring the epigenetic landscape of renal cancer. For example, scCancerExplorer is a comprehensive database that allows for the exploration of single-cell genomics, epigenomics (chromatin accessibility and DNA methylation), and transcriptomics data across various cancer types, including renal cancer. This database provides several modules and features for multi-omics cancer studies, including functionalities for analyzing single-cell epigenomes, such as DNA methylation plots, epigenomic browsers, and tools for TF analysis. These resources provide valuable insights into epigenetic regulation and transcription factor research in ccRCC and promote further exploration in related fields [274]. Wilms tumor, a type of kidney cancer primarily affecting children, has been associated with mutations in the histone acetylation reader AF9 family transcriptional elongation regulator (ENL) [275]. scRNA-seq and scATAC-seq on mouse kidneys have revealed that *ENL* mutations lead to impaired kidney differentiation, resulting in undifferentiated cell structures akin to those found in nephroblastoma. Mutated *ENL* disrupts cellular differentiation programs during kidney development by reshaping the gene regulatory landscape, leading to developmental defects. It may also affect the interaction between stromal and nephron progenitor cells, particularly by upregulating WNT5a in Forkhead box (Fox)D1 + stromal progenitor cells, influencing the WNT signaling pathway in nephron progenitor cells and contributing to abnormal kidney development [276]. These findings suggest that *ENL* mutations alter normal kidney gene expression regulation, disrupt the TF regulatory network, and may be involved in the development of Wilms tumor.

TFs typically exert control over gene expression at the transcriptional level, and alterations in their regulatory networks have significant implications in the onset and progression of cancers [277–282]. Crucially, a thorough comprehension of TFs in cancers contributes significantly to the development of precision treatment strategies [283–287]. In ccRCC, scATAC-seq analysis comparing cancer cells with normal PT cells has revealed that the activator protein-1 (AP-1) family of TFs exhibits stronger binding affinity for DNA in ccRCC cells. FBJ murine osteosarcoma viral oncogene homolog (FOS) and Jun proto—oncogene (JUN) family genes, components of AP-1, show enhanced expression in ccRCC cells and their associated target genes are enriched in various pathways relevant to tumorigenesis and progression. This suggests that the AP-1 family of TFs plays a vital role in ccRCC development and progression. Integration of scRNA-seq and scATAC-seq analyses has identified a set of TFs highly expressed and specifically enriched in ccRCC tumors, including orthopedia (OTP), ventral anterior homeobox (VENTX), homeobox C5 (HOXC5), and insulin gene enhancer binding protein 1 (ISL1). These TFs form a regulatory network governing the expression of multiple tumor-specific genes, and their knockdown significantly inhibits tumor cell proliferation and promotes cell death [288, 289]. TFs in ccRCC exhibit cell type-specificity across different cellular components. Multi-omics studies have identified cell type-specific TFs, such as zinc finger E—box—binding homeobox (ZEB1) in MCs, spleen—focus—forming virus (SFFV) proviral integration oncogene spi—C (SPIC) in B cells, eomesodermin (EOMES) in NK cells, ETS variant transcription factor 4 (ETV4) in non-tumor cells, E—twenty—six (ETS) transcription factor 1 (ETS1) in T cells, early B—cell factor 2 (EBF2) in cancer-associated fibroblasts (CAFs), and SRY—related HMG—box 8 (SOX8) in ECs. Furthermore, TF footprint analysis has revealed tumor cell-specific binding TFs, including hepatocyte nuclear factor (HNF) family members, TEA domain transcription factor 3 (TEAD3), and nuclear factor I B (NFIB). Among these TFs, HNF1B, which is associated with developmental kidney diseases, may provide new insights into ccRCC pathogenesis [255]. In terms of nccRCC, scRNA-seq has identified upregulation of genes involved in lipid metabolism, regulated by transcription factors like CCAAT/enhancer—binding protein beta (CEBPB), Kruppel—like factor (KLF)6, and KLF4. This aberrant expression may affect lipid metabolism in tumor cells, contributing to tumorigenesis and progression. In pRCC, the expression of the autophagy-related TF nuclear receptor subfamily 1 group H member 4 (NR1H4) is downregulated compared to normal PT, whereas in CDRCC cells, NR1H4 is upregulated. This indicates heterogeneity in autophagy regulation among nccRCC subtypes, affecting nutrient utilization, stress response, and TME interactions. Other TFs associated with ccRCC, such as retinoic acid receptor alpha (RARA), signal transducer and activator of transcription 3 (STAT3), nuclear factor kappa—light—chain—enhancer of activated B cells (NFκB), and POU domain class 2 transcription factor 2 (POU2F2), are also upregulated in nccRCC. NFκB upregulation may help MCs evade immune surveillance by inducing T cell exhaustion and suppressing macrophage function. STAT3 upregulation may relate to MCs proliferation, survival, and immune escape. Abnormal POU2F2 and RARA expression may influence MCs differentiation and biological properties [213].

These studies shed light on the importance of epigenetic regulations, TFs, and their networks in renal cancer development and progression, providing valuable insights for understanding the underlying biology and potential therapeutic strategies.

#### Single-cell sequencing technology decodes the complex cell-cell communication networks in renal cancer

The complex cell–cell communication within the TME is closely associated with tumor development and therapeutic response [290–294]. Fortunately, SCS has elevated the study of cell–cell communication to a higher resolution and precision, enabling researchers to gain a clearer and more comprehensive understanding of these interactions [295–299]. In ccRCC, a subset of tissue-resident macrophages (TR Mac) expresses interleukin-1β (IL-1β), which induces the expression and activation of CEBPB, a TF responsive to inflammatory signals in MCs. This leads to the expression of matrix metalloproteinases (MMPs) and promotes invasion and epithelial-mesenchymal transition (EMT) in ccRCC [150, 300]. MCs secrete MIF, which acts on TAMs via receptors CD44 and CD74, promoting ccRCC progression, angiogenesis, and immune evasion. Tregs secrete lymphotoxin beta (LTB), engaging with lymphotoxin beta receptor (LTBR) on MCs and influencing tumor progression. High expression of LTB and LTBR correlates with poorer clinical outcomes in ccRCC [288]. Interestingly, intercellular interactions between tumor ECs and myeloid cells, such as macrophages, are the strongest observed in ccRCC. The specific mechanisms underlying these interactions require further investigation, but certain molecules like C3 and its receptor C3AR1 are involved [130]. von Hippel—Lindau *(VHL)* gene mutations impact intercellular communication patterns in ccRCC, and different pathways show reduction or altered cell–cell communication in the absence of *VHL* mutations. Molecules associated with cell–cell communication, such as CD27, FASLG, and granzyme (GZM) A, correlate with CD8 + T cell infiltration and differentiation, suggesting their role in T cell responses [301].

In addition to immune cells, renal cancer cells engage in complex intercellular communication with stromal cells. For example, ccRCC.mes cells induce the transition of pericytes to myofibroblasts through secretion of transforming growth factor – β (TGF-β), IL6-IL6 receptor (R), and growth arrest-specific protein 6 (GAS6) – AXL receptor tyrosine kinase (AXL), as well as pyruvate dehydrogenase kinase (PDK)1-PDK2. Reciprocally, myofibroblasts promote EMT in ccRCC.mes cells through secretion of TGF-β, GAS6-AXL, and CXCL12-CXCR4 pathways, forming positive feedback signaling network [131]. Another study identified significant communication between MCs and a subset of myofibroblasts, potentially promoting tumor growth, invasion, and metastasis through the myelin protein zero (MPZ) signaling network [199].

**Fig. 3:**
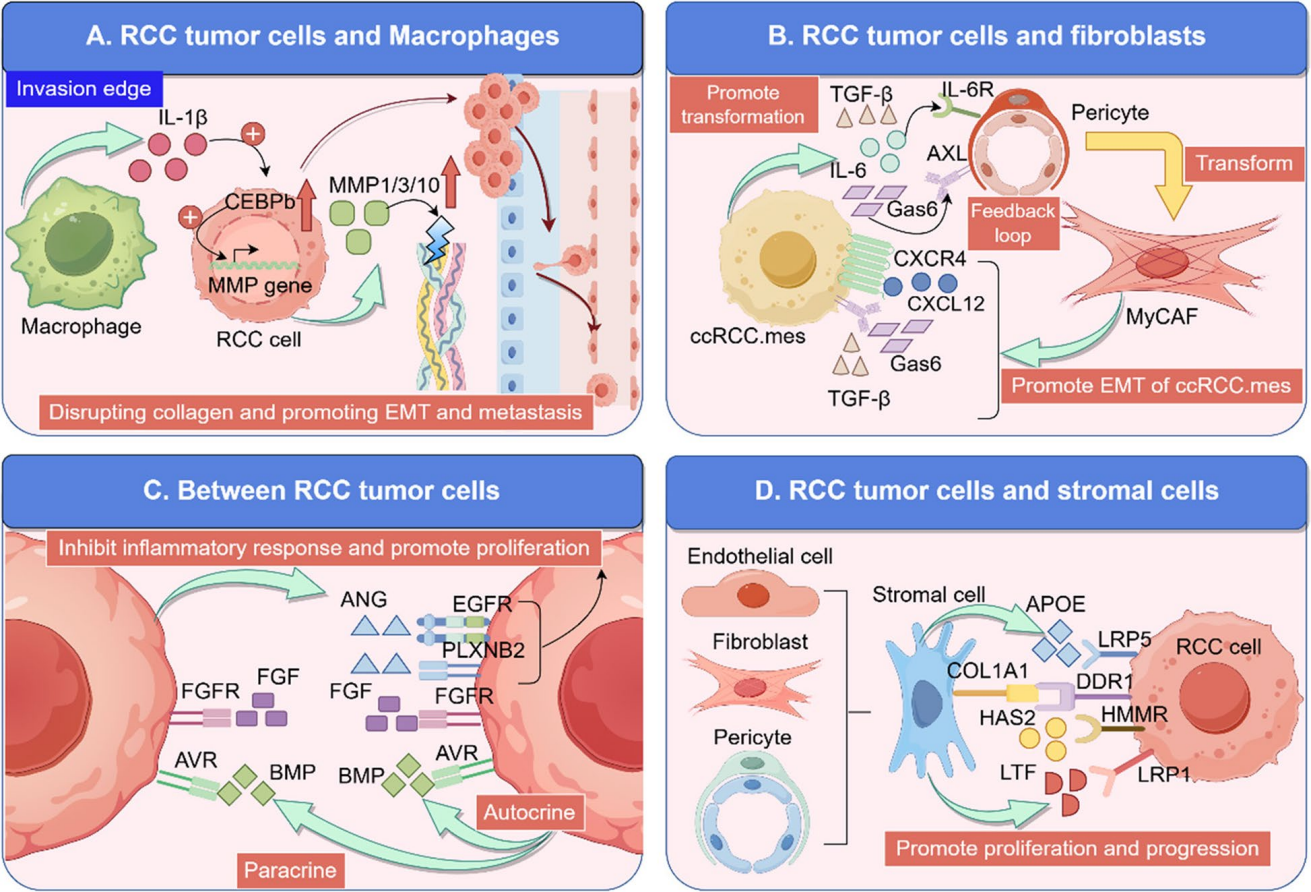
The complex cell–cell communication networks in renal cancer illuminated by single-cell sequencing. **A** In RCC, the interaction between tumor cells and macrophages plays a critical role in mediating the degradation of the ECM and the promotion of the RCC EMT and metastasis. **B** In ccRCC, mesenchymal-like cells (ccRCC.mes) within the TME drive the differentiation of pericytes into myofibroblasts (myCAF). Conversely, these myofibroblasts enhance the mesenchymal characteristics of ccRCC cells, thereby promoting the EMT process. **C** RCC tumor cells engage in intricate intercellular communications, with the ANG-EGFR/PLXNB2 pathway playing a pivotal role in suppressing inflammatory responses and promoting tumor cell proliferation. **D** There is extensive intercellular communication between RCC tumor cells and stromal cells, ultimately contributing to the advancement of the RCC. This figure was created based on the tools provided by Figuredraw.com

Additionally, significant cell–cell communication exists among MCs in ccRCC. Analysis through scRNA-seq has revealed increased expression of angiogenin (ANG) and its receptors EGFR and Plexin-B2 (PLXNB2) in MCs of ccRCC. Notably, the ANG signaling pathway downregulates inflammatory responses and promotes the proliferation of MCs [302]. Furthermore, Ghoshdastider et al. conducted a single-cell analysis of intercellular communication across various cancers, including ccRCC and pRCC, and found that MCs in ccRCC and pRCC are enriched with bone morphogenetic protein (BMP) ligands and activin A receptor (ACVR) receptors, fibroblast growth factor (FGF) signaling pathways (involving various FGF ligands and receptors), and Eph/ephrin signaling pathways through autocrine or paracrine mechanisms. Interactions between stromal cells and MCs are primarily mediated through pathways such as APOE-LRP5, collagen type I alpha 1 (COL1A1)- discoidin domain receptor 1 (DDR1), hyaluronan synthase 2 (HAS2)- hyaluronan—mediated motility receptor (HMMR), inhibin beta A (INHBA)-ACVR2B, and lactotransferrin (LTF)-LRP1 [303]. These cell–cell communication networks are summarized in Fig. 3.

### Single-cell sequencing technology illuminates the intricate panorama of tumor immune microenvironment in renal cancer

Numerous studies have demonstrated that in the renal cancer TME, the proportion of immune cells significantly outweighs that of tumor cells, underscoring the critical importance of the TIME in renal cancer [255, 288].

#### CD8 + T cell

Research indicates that CD8 + T cells constitute a predominant immune cell population within the RCC TIME, accounting for an average of 51%, and play a crucial role in the TIME [304]. CD8 + T cell exhaustion is linked to tumor progression, immune evasion [305, 306] and immunotherapy resistance [307, 308]. ScRNA-seq and scTCR-seq of T cells across various cancers have revealed that CD8 + T cells in RCC exhibit greater diversity compared to normal tissues, with the presence of multiple CD8 + T cell subsets. Notably, among these, exhausted CD8 + T cells are relatively more common [309]. The scRNA-seq analysis of ccRCC tumors and adjacent non-tumor tissues has been conducted, revealing a higher abundance of exhausted CD8 + T cells within the tumor tissue compared to the adjacent normal tissue [175, 254]. Additionally the terminally exhausted CD8 + T cells are more common in metastatic, advanced stages [225], tumor-normal boundary and high-grade (HG) tumor within ccRCC [207]. As ccRCC advances, CD8 + T cells increasingly express inhibitory checkpoints like programmed cell death protein 1 (PD-1), TIM-3, and lymphocyte—activation gene 3 (LAG3), and TFs thymocyte selection—associated high mobility group box protein (TOX) and EOMES, which are associated with terminal exhaustion [225, 254]. This suggests progressive CD8 + T cell exhaustion with disease advancement. Additionally, terminally exhausted CD8 + T cells have significantly reduced T—cell receptor (TCR) diversity compared to adjacent normal tissues, indicating that the exhausted state may be linked to decreased TCR abundance [225]. Moreover, the diversity of the T cell receptor beta chain (TCRB) is positively correlated with the numbers of naïve CD8 + and CD4 + T cells, indicating that a reduction in TCRB diversity is associated with an unfavorable immune state [310]. Pseudotime trajectory analysis demonstrated a transition in CD8 + T cells from cytotoxic to pre-dysfunction and dysfunction states. This transition is characterized by the downregulation of cytotoxic genes, including killer cell lectin-like receptor G1 (KLRG1), granulysin (GNLY), and GZMH, alongside the upregulation of dysfunction-related genes such as cytotoxic T-lymphocyte-associated protein 4 (CTLA4), hepatitis A virus cellular receptor 2 (HAVCR2), and LAG3. Pre-dysfunction-related genes, specifically CXCR4, GZMK, and GZMA, exhibit an initial increase followed by a decrease, indicating a pathway toward functional exhaustion in CD8 + T cells. Additionally, scTCR-seq revealed that CD8 + T cell expansion and tissue localization significantly influence their exhaustion levels [150]. In terms of metabolism, the activity of energy metabolism-related pathways initially increases, then decreases, and subsequently rises again, while amino acid metabolism exhibits distinct trends [207]. Raghubar et al. conducted a comprehensive study of CD8 + T cells in ccRCC using spatial transcriptomics sequencing (ST-seq) along with published scRNA-seq and TCR-seq datasets. Their findings demonstrated variations in the distribution of CD8 + T cells across different grades of ccRCC TIME, noting a higher presence of CD8 + tissue-resident cells in low-grade (LG) TIMEs compared to the highest proportion of exhausted proliferating CD8 + cells in HG TIMEs. Gene expression analysis revealed that certain subsets of exhausted CD8 + T cells within HG TIMEs express the stem cell-like progenitor gene transcription factor 7 (*TCF7*), whereas some proliferative CD8 + T cell subsets express the immunomodulatory gene ectonucleoside triphosphate diphosphohydrolase 1 (*ENTPD1*) [311]. Regarding gene expression regulation, several TFs—including EOMES, basic leucine zipper transcription factor ATF-like (BATF), and multiple Rel/NF-κB family transcription factors—are highly enriched in the exhausted cell cluster [288]. Additionally, TFs such as positive regulatory domain zinc finger protein 1 (PRDM1), nuclear receptor subfamily 4 group A member 1 (NR4A1), and TOX are also found to be enriched within this cluster [131]. Furthermore, the process of exhaustion is marked by the upregulation of four members of the nuclear factor of activated T-cells (NFAT) family, the downregulation of AP-1, and the enrichment of the human immunodeficiency virus type I enhancer-binding protein (HIVEP) family [195]. Additionally, Mujal et al. discovered a relationship between the status of CD8 + T cells and the levels of Tregs and macrophages. In patients with elevated ratios of macrophages to monocytes and high frequencies of Tregs, CD8 + T cells exhibited signs of exhaustion. They displayed increased expression of exhaustion markers PD-1 and CD38, along with a diminished ability to proliferate, as indicated by Ki-67 levels. Conversely, patients with lower ratios of macrophages to monocytes and robust infiltration of conventional type 1 dendritic cells (cDC1) showed CD8 + T cells with reduced expression of exhaustion markers and elevated levels of the checkpoint molecule CTLA-4, suggesting a state of ongoing activation [240]. These discoveries align with the work of Kersten and colleagues, which establishes a close relationship between the exhaustion of TAMs and CD8 + T cells [312]. Furthermore, scATAC-seq has identified eight distinct CD8 + T cell subsets in ccRCC, each with unique chromatin accessibility and cellular states. CD8 + T cells isolated from 4 clusters, predominantly from tumor tissues, show higher accessibility to genes linked to dysfunction. During CD8 + T cell dysfunction progression, chromatin states undergo heterogeneous changes, indicating complex interactions between epigenetic modifiers. CD8 + T cell dysfunction also involves reshaping the regulatory landscape of stress response genes, offering an epigenetic insight into the mechanisms of CD8 + T cell dysfunction [313]. Interestingly, while NF-κB typically plays a crucial role in T cell activation [314–316], it was observed that in advanced dysfunctional CD8 + T cells, NF-κB promoted apoptotic programs. Furthermore, high expression levels of NF-κB were associated with more advanced disease stages and poorer overall patient survival in ccRCC, suggesting that NF-κB may influence the TME and patient clinical outcomes by inducing apoptosis in dysfunctional CD8 + T cells [313]. In addition, Xu et al. found that the expression of proline-yielding enzyme PYCR1 was negatively correlated with the proportion of GZMB + CD8 + T cells, suggesting that PYCR1 may inhibit the cytotoxicity of CD8 + T cells. Moreover, samples with high PYCR1 expression exhibited increased PD-1 + CD8 + T cells and reduced tissue-resident memory T (TRM) cells, indicating that PYCR1 might contribute to CD8 + T cell exhaustion and impact tumor immune surveillance and immune memory functions [317]. Ning et al. observed a higher incidence of RCC in males compared to females, with male patients presenting at more advanced pathological stages. Immunotherapy appeared to be more beneficial for male patients in terms of progression-free survival (PFS). Analysis using scRNA-seq data revealed a significantly higher proportion of terminally exhausted CD8 + T cells in tumors of male RCC patients compared to female patients. Additionally, the androgen-androgen receptor axis appeared to play a crucial role, suggesting that androgens promote the exhaustion of CD8 + T cells, thereby fostering an immunosuppressive environment [318]. This observation is consistent with another study that found a correlation between androgen receptor activity and impaired functionality of CD8 + T cells in cancers [319]. In nccRCC, which includes subtypes such as pRCC, chrRCC, CDRCC, and sarcomatoid RCC (sarRCC), a study by Chen et al. utilizing scRNA-seq revealed that CD8 + T cells in nccRCC tumors exhibit an exhausted state, similar to ccRCC. Interestingly, the number of CD8 + LAG3 + T cells in tumor tissues was higher compared to peritumoral tissues, and their presence was associated with poor prognosis. This suggests that the exhausted state of CD8 + T cells is consistent across different nccRCC subtypes and may serve as a potential indicator for assessing patient prognosis in nccRCC [213]. Similar to ccRCC, in FHRCC, CD8 + T cells can be classified into distinct subtypes that undergo dynamic shifts from a naive state to a terminally exhausted state during tumor progression, accompanied by significant changes in gene expression. Cluster 3 in the terminal phase exhibits high expression of genes related to apoptosis and glycolysis, while cluster 2 shows downregulation of genes associated with immune activation. Notably, even with treatment using ICB, exhaustion-related genes continue to be upregulated, and the infiltration of CD8 + T cells in the TIME is not associated with treatment response. This suggests that ICB therapy may face challenges in reversing the terminal exhaustion of CD8 + T cells in FHRCC [242]. However, scRNA-seq analysis of tumors from RCC pancreatic metastasis (RCCpm) revealed an intriguing finding: CD8 + T cells in RCCpm exhibit an active cytotoxic state, expressing GZMK [320], which contradicts the conventional understanding that CD8 + T cells in metastatic tissues are often exhausted [225]. This suggests that while actively cytotoxic CD8 + T cells may have a tumoricidal effect, they may also be involved in the process of immune evasion.

CD8 + T cells play a crucial role in cancer immunotherapy [321–327]. scRNA-seq and spatial genomics have unveiled diverse interactions between T cell states and other cell types in advanced ccRCC under ICB therapy. In ICB responders with low high human leukocyte antigen (HLA) peptide binding affinity, pre-exhausted CD8 + T cells interact with pro-inflammatory tumor-associated macrophages (TAMs) and paired box gene 8 (PAX8) + MCs, while exhausted CD8 + T cells interact more with pre-exhausted CD8 + T cells, ECs, and MCs. In non-responders with high HLA peptide binding affinity, both CD8 + T cell subsets primarily interact with anti-inflammatory CD206-high TAMs. Cell–cell communication analysis has revealed differences among CD8 + T cell subsets between ICB responders and non-responders, with Myxovirus resistance 1 (MX1)-high CD8 + T cells and circulating CD8 + T cells playing more significant roles in communication in responders, elucidating the functions and mechanisms of T cells in the ccRCC immune microenvironment [328]. scRNA-seq has identified distinct CD8 + T cell clusters, including tissue-resident and NK-like T cells. In ICB-responsive and refractory patients, the most expanded clonotypes originate from CD8A + tissue-resident clusters. ICB responders show enrichment of these clusters across tumors, while resistant patients show T cell exclusion. Complete responders may exhibit a trajectory between tissue-resident and NK-like T cells, whereas refractory patients have relatively"fixed"tissue-resident T cells. This indicates the crucial role of tissue-resident T cells in antitumor immunity, with CD8A + tissue-resident T cell expansion post-ICB therapy correlating with a favorable response. The interconversion between tissue-resident, NK-like, and exhausted T cells may be linked to ICB therapy response [329]. Single-cell transcriptomic studies of advanced RCC during ICB therapy have demonstrated significant changes in CD8 + T cells pre- and post-treatment. In ICB-exposed patients, PD-1, TIGIT, and HAVCR2 are notably upregulated in the 4—1BB ligand—low expression (4-1BB-Lo) subset, particularly in responders. This subset also exhibits increased expression of effector molecules like GZMB, perforin 1 (PRF1), and interferon gamma (IFNG) and differentiation towards terminal exhaustion, indicated by the downregulation of genes linked to progenitor exhaustion. These findings suggest ICB therapy reshapes CD8 + T cells in advanced RCC, with the 4-1BB-Lo subset showing significant activation and differentiation towards terminal exhaustion in responders, providing insights into ICB resistance mechanisms in advanced RCC [226]. Additionally, expanded TCR clones in responders show higher CD137 expression and upregulation of GZMK. Non-responders have lower TCR clonality before treatment, and post-treatment, expanded TCR clones are often replaced, indicating a dynamic TCR replacement process. These findings offer insights into the mechanisms of response to ICB in ccRCC and suggest potential targets for developing more effective immunotherapies [330]. Traditionally, RCC is considered radioresistant [331–336]. However, Chow et al. investigated the changes in the TIME of RCC before and after radiotherapy using scRNA-seq and spectral flow cytometry. They found that radiotherapy increased the levels of effector and proliferating CD8 + T cells, enhanced MC antigen presentation, and improved interactions with CD8 + T cells. These findings reveal the contributions of both immune and tumor-intrinsic factors in radiation-induced tumor immunogenicity, providing a theoretical basis for optimizing combined radiotherapy and immunotherapy strategies [337].

CD8 + T cells display heterogeneity across various types of cancer, highlighting the importance of understanding their diversity in cancer research [338–344]. Particularly in renal cancer, scRNA-seq has revealed significant heterogeneity within CD8 + T cells in the TIME. Distinct PD-1 + subsets express varying levels of co-inhibitory receptors (Tim-3), activation markers (CD38, HLA-DR), and co-stimulatory receptors, such as inducible costimulator (ICOS) and 4-1BB. The T-0 subset, with the highest PD-1 expression, exhibits strong positivity for co-inhibitory receptors, activation markers, and co-stimulatory receptors, but low CD127 levels. These findings imply that targeting PD-1 may be more effective than targeting a single inhibitory receptor in ccRCC treatment [304]. The Study have indicated that the positivity rate for PD-1 expression in CD8 + T cells within the TIME of ccRCC is 21.9%, further emphasizing the significant role of CD8 + T cells in immunotherapy [255]. As identified through scRNA-seq and scTCR-seq. Beyond typical functional states (naive, effector, memory, pre-dysfunction, and dysfunction), high expression of tissue residency markers (ITGAE, CD69), specific CXCL13 expression in TRM cells, and novel gamma-delta T cell clusters were observed [150]. Furthermore, tissue-resident CD8 + T cells have been categorized into two subsets, one highly expressing genes associated with naive/memory phenotypes and the other characterized by high expression of effector molecules [288]. Additionally, a subgroup of tissue-resident CD8 + T cells exhibit characteristics of progenitor exhaustion, while another subgroup displays higher cytotoxicity features [131]. Zhang et al.'s research suggests that the previous notion that CD8 + T cells alone predict poor outcomes is not accurate. They have classified CD8 + T cells into distinct subgroups and found that high infiltration of 4-1BB + T cells or NKT cells is associated with poorer survival, whereas high infiltration of effector-exhausted T cells is linked to better survival. This indicates that a comprehensive set of markers is necessary for assessing the prognosis of ccRCC [130]. scRNA-seq studies on TT in ccRCC reveal distinct CD8 + T cell subsets between TT and PT. TT contains more tissue-resident CD8 + T cells with a progenitor exhaustion phenotype. CD8 + T cell trajectories in both PT and TT converge towards exhaustion. However, a subset in TT shows progenitor exhaustion traits linked to immunotherapy responses and better prognosis, suggesting these traits could predict immunotherapy response in ccRCC patients [176]. In terms of spatial heterogeneity, the proportion and subset distribution of CD8 + T cells vary across tumor regions in different ccRCC patients, with some areas containing up to 80% cytotoxic CD8 + T cells [growth—related oncogene B (GRB), CD57 +]. CD8 + T cells are also involved in the formation of specific cellular neighborhoods, such as an enriched T cell domain in certain tumor regions. Furthermore, CD8 + T cells are associated with the expression of cytokines and are linked to cytokines such as CCL4 and CCL5 within immune-inflammatory cellular neighborhoods [257]. ST-seq, notably Decoder-seq, has categorized renal cancer cells into distinct CN, revealing CD8 + T cells are sparser in the tumor core, likely due to an immune-suppressive environment, and more common in the infiltrative margins where immune responses are more active [123]. Through the integration of scRNA-seq with multi-omics technologies, ccRCC has been categorized into four distinct immunological subtypes (IMs). Analysis has shown variability in immune cell clusters and tumor metabolism among these IMs. Notably, one IM is marked by an abundance of CD8 + T cells, terminally exhausted T cells, and increased pyrimidine metabolism, which is associated with higher CD8 + T cell infiltration and poor prognosis. This suggests that CD8 + T cell function may be influenced by the metabolic state of the tumor and vice versa. In contrast, other IMs have fewer CD8 + T cells [190]. Using single-cell long-read sequencing (scNanoRNAseq) to study the TIME in RCC, differential expression of transcript isoforms (DCIs) has been found to shape cell functions and interactions. For example, myeloid cells and T cells both express HAVCR2, but their most dominant transcripts (MDTs) differ. This variation likely assigns them distinct roles in immune responses, thereby influencing the immune status and intercellular interactions within the TME [345].

#### CD4 + T cell

CD4 + T cells exhibit a spectrum of heterogeneity within the TME in various cancers [346–349], including renal cancer. In the TME of renal cancer, CD4 + T cells demonstrate significant heterogeneity across different aspects, reflecting the complexity of the TME. Regarding the phenotypic aspects of cells, multi-regional genomic and single-cell transcriptomic sequencing in renal cancer has identified subsets of CD4 + T cells, such as CD4 + naive/central memory T cells and CD4 + Tregs, revealing their distinct tissue distribution. CD4 + T cells also exhibit complex interactions with other immune cells. In the context of ICB therapy, changes in CD4 + T cell status and function correlate with treatment response, indicating their potential as predictive biomarkers for immunotherapy efficacy [150]. Similarly, analysis of multi-regional scRNA-seq data in ccRCC has also categorized CD4 + T cells into multiple subsets, including CD4 + effector, CD4 + proliferating, and CD4 + activated immediate early gene (IEG) T cells, extending beyond CD4 + naive T cells and CD4 + Tregs [329]. A PD-1 + subset within CD4 + T cell populations, co-expressing CD38 and Tim-3, has also been identified, highlighting a unique phenotype that may have implications for immune response and treatment strategies [304]. scATAC-seq reveals specific tissue distribution in CD4 + T cell subsets. The C1-CD4 subset, primarily from peripheral blood, shows high chromatin accessibility for naive marker genes like lymphoid enhancer—binding factor 1 (*LEF1*) and selectin L (*SELL*). The C3-CD4 subset, mainly from tumor tissues, exhibits high accessibility to genes such as *IL7R* and *IL2*, linked to memory-like and/or effector-like fates, and shows increased accessibility to follicular helper T cells (TFH)-specific genes like *IL21*. The C12-CD4 subset, also predominantly from tumor tissues, is associated with high chromatin accessibility of tumor-infiltrating Treg markers, including tumor necrosis factor receptor superfamily (TNFRSF)18, ICOS, and CTLA4 [313]. In terms of disease progression, CD4 + T cells are generally more abundant in normal tissues and early-stage ccRCC. However, certain subsets, such as activated CD4 + T cells and Tregs, are also present in significant proportions in later stages of the disease. This suggests a heterogeneity in their involvement throughout the progression of ccRCC [225]. In the context of cancer immunotherapy, although CD4 + T cells may not be as central as CD8 + T cells, they still hold significant value in the immune response against tumors [350–355]. Single-cell studies of the TME across various cancers have shown an enrichment of CD8 + stress-reactive (STR) cells and CD4 + TSTR cells in RCC patients following ICB therapy. Notably, CD4 + TSTR cells were significantly enriched in non-responders, and the expression of heat shock protein genes heat shock protein family A member 1 (HSPA1) A and HSPA1B was markedly upregulated in T cells. This suggests that STR cells may modulate immune responses in RCC through changes in the expression of heat shock protein genes and may be associated with resistance to immunotherapy [356, 357].

Tregs, a subset of CD4 + T cells, often play an immunosuppressive role within the TME across various types of cancer and their activity is associated with cancer progression [358, 359] and resistance to immunotherapies [360–362]. Studies using scRNA-seq and scTCR-seq across different cancers have revealed that TNFRSF9 + Tregs are the most abundant population in RCC, with a significantly higher frequency in tumor tissues compared to normal tissues and blood [309]. ScRNA-seq analysis of ccRCC has demonstrated that Tregs are more abundant within tumor tissues [227] and metastatic tissues [363], whereas CD4 + T cells are more prevalent in normal tissues [227]. Moreover, Hu et al. found that Tregs are highly enriched almost exclusively in tumor tissue, implying their significant role in the TIME of ccRCC [195]. Interestingly, PD-1-positive CD4 + T cells are also enriched in tumor tissues and present in metastatic tissues, suggesting their potential role in disease progression [304]. Additionally, Liu et al. observed significant activation of Tregs in the high expression group of malignant epithelial cell-related genes (*MECRGs*) in ccRCC, indicating that *MECRGs* promote tumor immune suppression [241]. ScRNA-seq analysis of Treg cells has revealed unique transcriptional profiles of tumor-infiltrating Treg (TI Treg) cells in ccRCC compared to peripheral blood (PB) Treg cells. The transition from PB to TI Treg cells follows two divergent paths, Cell Fate #1 (CF1) and Cell Fate #2 (CF2). CD177, a surface protein marker, is associated with the more suppressive CF1 TI Treg cells. Experimental evidence has confirmed CD177's significant inhibitory effect on effector CD4 + T cells, and blocking CD177 with anti-CD177 antibodies abolishes this suppression. Treg cell-specific CD177 deficiency affects tumor growth and the number of TI Treg cells, highlighting the heterogeneity of TI Treg cells and the critical role of CD177 in their immunosuppressive function [364]. In scRNA-seq studies of TT in ccRCC, it was found that the proportion of CD4 + T cells in the PT is lower than that in the adjacent normal renal tissue (ART) and TT. In contrast, the proportion of Tregs is higher in the PT. These findings suggest that the primary tumor may be in a state of stronger immunosuppression [176]. Furthermore, a study has found that Tregs accumulate in samples with high PYCR1 expression, indicating that PYCR1 may promote tumor immune suppression [317]. Regarding immunotherapy, scRNA-seq and spatial omics studies on the TME in advanced ccRCC patients have shown that Tregs are key components involved in significant crosstalk with other immune cells within a complex network. Comparisons between responders (partial response, PR) and non-responders (stable/progressive disease) to ICB therapy have revealed differences in intercellular communication involving Tregs, suggesting their influence on response to ICB therapy and potential impact on tumor cell sensitivity or resistance to ICB [328]. The proportion of Treg cells in the TME of responders is lower than that in non-responders, indicating that Treg cells may contribute to resistance to ICB therapy [330]. In preclinical studies, combinations of therapies such as anti-IL-1β and cabozantinib have been shown to enhance CD4 + effector T cell responses and increase naive T cells in tumor-draining lymph nodes [365]. Another study identified significant cytotoxic CD4 + T cells in metastatic RCC (mRCC), distinguishing between CD4 + GZMK + T cells with lower cytotoxic potential and CD4 + GZMB + T cells with higher cytotoxicity. Patients with higher levels of CD4 + CTLs in the ICB treatment group showed better overall and PFS, indicating the potential of CD4 + CTLs as predictive biomarkers for ICB treatment response in mRCC patients [366]. There is also a correlation between the CD8 + exhausted T cell population and the CD4 + Treg population, suggesting their collaboration in modulating tumor immune responses [329]. Additionally, an increase in Treg cell activity within tumor tissues is associated with exhaustion of the CTL-1 subset within CD8 + T cells [175]. Reticulocalbin-1 (RCN1) has been found to be highly expressed in ccRCC tumor cells and associated with poor prognosis. SCS analysis reveals an interaction between Tregs and RCN1, which is linked to poor outcomes and activation of inflammatory pathways in various cancers [367]. Interestingly, Treg cells also exhibit differences across different subtypes of RCC. Decoder-seq analysis has shown a higher abundance of Tregs in chrRCC compared to ccRCC, indicating a more immunosuppressive TME in chrRCC. In ccRCC, Treg cells interact with ECs, B-lineage cells, and MCs, while in chrRCC, Tregs interact with fibroblasts, ECs, and myeloid cells [123].

#### Macrophage

It has been reported that TAMs play a significant role in various cancers progression [368–370] and also have implications for immunotherapy [371–375], including renal cancer [376]. A recent scRNA-seq study revealed the complex phenotypic landscape of macrophages within RCC tumors, surpassing the classical M1/M2 dichotomy [377, 378]. Distinct macrophage subsets show varying expression of immunomodulatory genes. Notably, ICB therapy induces a shift in some TAM towards a pro-inflammatory phenotype. However, this shift is accompanied by upregulation of immune checkpoint and anti-inflammatory signaling genes, which may lead to immune adaptation and therapy resistance [226]. Research utilizing scRNA-seq and spatial omics technologies has identified various macrophage subpopulations, including pro-inflammatory M1 and immunosuppressive M2 macrophages, with altered proportions and phenotypes in the TME. Subsets like v—set immunoregulatory receptor (VSIR)-high TAMs are more prevalent or active in responders to ICB therapy. Macrophages interact complexly with immune cells such as CD8 + T cells through antigen presentation, co-stimulation, and signaling pathways, impacting immune responses. These interactions correlate with outcomes of ICB therapy, where a higher proportion of pro-inflammatory macrophages may enhance patient response to treatment, highlighting the crucial role of macrophages in RCC tumor immunity [328]. Clinical signatures tied to macrophages are strongly linked to patient prognosis and responses to treatments like immune ICB and tyrosine kinase inhibitors (TKIs). The myeloid inflammatory signature correlates with TKIs therapy outcomes, while the interferon—stimulated gene (*ISG*)-high TAMs subset characteristics are associated with PFS after sunitinib treatment. Additionally, TAMs and interactions with CD8A + tissue-resident T cells show distinct patterns with ICB therapy: inhibitory interactions are predominant in resistant patients, whereas pro-inflammatory interactions occur in responsive patients [329]. In a large-scale pan-cancer multi-omics study, M1 macrophage presence strongly correlates with responsiveness to ICB therapy in renal cancer, being more prevalent in responsive patients. In responders, M1 macrophages exhibit immune activation gene expressions, including higher levels of interferon regulatory factor (IRF)1 and the release of chemokines CXCL10 and CXCL11, which attract T cells via CXCR3. There is also an imbalance in macrophage-T cell ligand-receptor interactions, particularly in the IFNG-IFNGR2 axis, creating a positive feedback loop that enhances their communication and impacts the efficacy of ICB therapy. These findings highlight the crucial role of macrophages and their interaction with T cells in the success of immunotherapy for renal cancer [379]. Studies have shown that treatments such as IL-1β blockade, ICB, or TKIs can reconfigure the myeloid compartment in the TME through T cell-independent mechanisms, enhancing pro-inflammatory M1-like TAMs and suggesting new immunotherapy strategies for RCC. For example, anti-IL-1β reduces certain subsets of TAMs and increases M1-like TAMs, while cabozantinib alone or in combination with anti-IL-1β boosts M1-like TAMs and reduces M2-like TAMs [365]. However, it is worth noting that pro-inflammatory TAMs in ccRCC express genes associated with tumor metastasis, indicating their complex role in this type of cancer [156]. These findings provide crucial insights into the TME of ccRCC and the mechanisms of immunotherapy.

Macrophages in RCC exhibit pronounced heterogeneity. Multi-region scRNA-seq has identified four distinct TAM subgroups in ccRCC, each with unique molecular profiles and immune phenotypes. The distribution of these TAM subgroups across patients and tissue regions correlates with the TIME and pathological features. These TAM subgroups interact with CD8 + T cells, CD4 + T cells, and dendritic cells (DCs), forming a complex network that significantly influences the tumor immune status [329]. Mass cytometry and PhenoGraph clustering algorithms have identified 17 distinct TAM phenotypes, with the subgroup 5 macrophage linked to immunosuppressive T cell populations, suggesting a role in modulating T cell functions through various molecules. Frequencies of TAM subgroup 11, 13, and5 have correlated with progression-free survival, indicating their potential as prognostic biomarkers for ccRCC patient outcomes [304]. M2-like macrophage subsets express markers such as CD68, CD163, and TREM2, and have immunosuppressive effects through immune checkpoint molecules, such as fibrinogen—like protein 1 (FGL1), PD-L2 and PD-L1, thus promoting ccRCC progression [195]. Moreover, sphingolipid metabolism is closely linked to M2 macrophage polarization [207]. In brain metastases of RCC, scRNA-seq data revealed a reduced expression of M1-like markers (HLA-DR and CD127) in macrophages from metastatic samples, suggesting that a decrease in M1-like macrophages may contribute to the progression of RCC metastasis [380]. Zhang et al. identified two macrophage subgroups in renal cancer. Subgroup A exhibits higher expression of pro-inflammatory genes (*IL1B*, *IL23A*, *CCL4*, *CCL3*, *CCL20*) and correlates with poor overall survival in ccRCC patients. Conversely, subgroup B's gene signature is linked to better survival outcomes [122]. Further research has revealed additional markers expressed by TAMs, such as glycoprotein nmb (GPNMB), macrophage scavenger receptor 1 (MSR1), and SLC40A1 and further research has revealed that TAMs can be divided into two subtypes, exhibiting heterogeneity in gene expression. One subtype highly expresses *CD86*, colony—stimulating factor 1 receptor (*CSF1R)*, and *CD163*, while the other expresses these genes at lower levels. Notably, SLC40A1 interacts with CP, a signature marker of ccRCC, as a ligand-receptor pair [255]. Mujal et al. classified macrophages in the RCC TIME into four subgroups: terminally differentiated C1q c chain (C1QC) + TAMs; TAMs with stress response programs, including selenoprotein P plasma 1 (*SPP1)*, which are less mature than C1Q + TAMs, showing higher monocyte marker levels like S100 calcium—binding protein A *(S100A)* genes, and lower major histocompatibility complex (MHC)-II gene levels; and TAMs expressing the antioxidant gene *SEPP1*, similar to C1Q + TAMs but enriched for folate receptor 2 (FOLR2) [240]. Zhang et al. utilized scRNA-seq to stratify macrophages into various subtypes and discovered that a high infiltration of C3AR1 + macrophages in tumors correlates with poorer survival, whereas a high infiltration of CD163 + macrophages or cDC2 is associated with better survival outcomes [130]. A study utilizing multi-region single-cell and spatial transcriptomics identified diverse macrophage subpopulations in RCC, including TAMs and TR Mac. TAMs include subsets like MHC-II + TAMs with distinct molecular profiles and tissue distribution, some showing pro-tumorigenic properties. The TR Mac.2 subset is enriched at the tumor-normal tissue interface, interacting with high-EMT MCs and promoting RCC progression. Conversely, the TR Mac.3 subset, enriched in the normal adrenal gland, exhibits significant M2 and phagocytic features. These findings highlight the phenotypic and spatial heterogeneity of macrophages in the TIME of RCC and their crucial role in disease progression [150]. Raghubar et al. refined TAM categorization in ccRCC into subtypes: TAM ISGint (intermediate interferon signaling gene expression, anti-tumor), TAM ISGhi (high interferon signaling gene expression), TAM HLAhi (high HLA-DR expression), TAM HLAint (intermediate HLA-DR expression), and tissue-resident monocytes (pro-tumorigenic). TAM ISGint cells are more prevalent in LG TMEs, while tissue-resident monocytes dominate HG TMEs. In HG-3 (a patient) TMEs, many pro-tumorigenic TAMs express the recurrence marker TREM2. In both HG-2 and HG-3 TMEs, TAM ISGhi lack immune suppressive markers HLA-G and cathepsin s (CTSS) [311]. Compared to adjacent normal tissues, ccRCC tumor tissues exhibit significant changes in macrophage number and distribution. Subsets like C1Q + TREM2 + macrophages are more abundant and closer to tumor cells, indicating a functional role related to their positioning. The density of C1Q + macrophages in tumor tissues is strongly linked to disease recurrence, suggesting that these macrophages are crucial in tumor recurrence and may help predict postoperative recurrence risk in ccRCC patients [227]. The gene expression and functions of C1Q + macrophages have been detailed, identifying three TAM subsets: TAM- lectin galactoside—binding soluble 3 (LGALS3) (immune suppression genes and M2 macrophage activation), TAM- regulator of cell cycle (RGCC) (pro-inflammatory), and TAM-C1QB (*APOE*, complement genes, and strong antigen presentation). TAM-LGALS3 primarily exhibits M2-like traits, TAM-RGCC mainly exhibits M1-like features, and TAM-C1QB has the highest phagocytosis score [131, 288]. The TF myocyte—enhancer factor (MEF)2C shows increased activity and expression in the TAM-C1QB subset, regulating multiple genes associated with macrophage functions. The target gene signature score correlates with better patient survival, suggesting a potential significant role for MEF2C [131]. scRNA-seq sub-cluster analysis identified macrophages with immunosuppressive (M2-like) traits overexpressing *TREM2* and *SPP1*, genes linked to tumor angiogenesis and ICB therapy. High TREM2 expression correlates with poorer survival in public spatial transcriptomic data from ccRCC, indicating TREM2 as a potential prognostic marker and immunotherapeutic target in ccRCC [175]. Guo et al. found that TAM in ccRCC exhibit high expression of the TF TCF7 ligand 2 (TCF7L2), which promotes M2 polarization and contributes to tumor immune suppression [381]. Compared to macrophages in TT, those in the PT exhibit a complex functional state, possessing both pro-inflammatory and immunosuppressive characteristics, which may facilitate immune evasion of tumor cells. In contrast, macrophages in TT play a significant role in pathways associated with ECM remodeling and tumor progression, promoting the growth of tumor thrombi by altering the TME [176]. Studies have focused on macrophages linked with the ECM in RCC to gain a deeper understanding. Coulton et al., using scRNA-seq analysis across various cancers, identified distinct TAM subsets in ccRCC. They discovered a notable subset, ECM + macrophages, characterized by high ECM-related gene expression (*COL3A1*, *COL1A1*, and *COL1A2*), potentially involved in ECM balance in the TME. This subset was enriched in therapy-unresponsive patients, unlike the IFNG + macrophages subset, which was prevalent in responsive patients and marked by high CXCL9 expression, a chemokine crucial for T cell recruitment to tumors and linked with ICB treatment response [382]. Targeting the ECM-modifying ECM + macrophages subset may enhance the efficacy of immunotherapies, while its predominance in non-responders may indicate an immune evasion strategy employed by the tumor [383]. To broaden the perspective on RCC heterogeneity, a study expanded its scope from within the tumor to encompass the entire tumor context. Using snRNA-seq, ccRCC was classified into distinct IMs, revealing macrophages with immunosubtype-specific traits. In IM3, a macrophage subset enriched in IFNG response was found, while IM4 was characterized by insulin-like growth factor (IGF)1 + macrophages associated with fibroblast accumulation. Macrophage-related gene expression differentiated immune and metabolic heterogeneity. IM3 exhibited changes in the pyrimidine metabolic pathway associated with CD8 + T cell infiltration, whereas IM4 showed tumor cell metabolic alterations related to macrophage functional changes. Macrophages significantly influenced tumor progression, and patients with IM4 had a poor prognosis. These macrophage-related changes impact tumor cell metabolism and regulation, providing insights into ccRCC biology and therapeutic strategies [190]. Macrophages in the TIME of various RCC subtypes are heterogeneous. Using Decoder-seq researchers identified distinct TAM distributions in ccRCC and chrRCC subtypes across different tissue regions. These macrophages interact variably with other cell populations within specific CN. During tumor progression, macrophages aid in tumor cell dissemination, and within specific CN, their interactions with immune cells influence cellular composition changes and EMT-related processes as tumor cells spread. Spatial gradient expression genes linked to macrophages in these CNs exhibit high expression of EMT-related genes, correlating with advanced RCC and poor prognosis, highlighting the potential importance of macrophage-associated features in RCC development and prognostic evaluation [123]. In a snRNA-seq research on RMC, similar to ccRCC, TAMs in RMC are part of the TIME, which includes MCs, CAFs, and immune cells. The tissue distribution of TAMs correlates with CAFs and RMC tumor cells, exhibiting distinct patterns. The study identified TAM subgroups, such as TAM1, TAM2, and TAM3, based on gene expression. TAM1 cells have a pro-inflammatory M1 phenotype, while TAM2 and TAM3 have an anti-inflammatory M2 phenotype, with TAM3 being the most pronounced. The shift in the M1/M2 distribution may affect the proliferative, invasive, and metastatic potential of tumor cells [144]. Chen et al., using single-cell genomics, categorized macrophages in nccRCC into five subgroups based on distinct origins: Normal-M with thrombospondin 1 (THBS1) +, sarRCC-M with ATP5F1E +, CDRCC-M with KRT8 +, chrRCC-M with SLC40A1 +, and pRCC-M with NADH dehydrogenase 1 alpha subcomplex 4—like 2 (NDUFA4L2) +. The Normal_M subgroup, derived from non-malignant tissue, expresses THBS1 and is linked to anti-angiogenic and tumor-suppressive activities. The chrRCC_M subgroup, expressing SLC40A1, is involved in inflammation. The CDRCC_M subgroup, marked by KRT8, facilitates communication with mesenchymal cells and macrophages during tissue repair. Notably, HAVCR2, an immune checkpoint molecule, is highly expressed in macrophages from chrRCC, pRCC, and sarRCC, indicating HAVCR2 as a potential therapeutic target in nccRCC [213].

Macrophages within tumors are associated with the exhaustion of CD8 + T cells [312, 384, 385]. As ccRCC progresses, there is a decrease in pro-inflammatory macrophages and an increase in anti-inflammatsory M2-like macrophages. Moreover, there is a bidirectional inhibitory interaction between terminally exhausted CD8 + T cells and M2-like macrophages, forming an immunological dysfunction circuit that further promotes the advancement of ccRCC [225]. VEGFA secretion by macrophages has also been observed in ccRCC, indicating their involvement in the intricate VEGF signaling pathway. This interaction with endothelial cells through receptors influences angiogenesis and vascular development, providing the tumor with nutrients and oxygen support, thereby facilitating tumor growth and progression [121]. Similarly, across various cancers, the expression of VEGFA by macrophages has been observed [386–388], further underscoring the significance of macrophages in the TIME.

#### NK cell

NK cells constitute a significant component of the TIME and exert complex functions in both tumor suppression and promotion processes [389–393]. NK cells in renal cancer exhibit heterogeneity. Utilizing scRNA-seq and spatial transcriptomics, distinct NK cell subsets have been identified, including the CD56 NK-1 subset [expressing CD44, chemokine (C—motif) ligand (XCL)1, XCL2, and killer—cell lectin—like receptor subfamily (KLR)C1], and the CD56 bright NK-2 subset [expressing FGF—binding protein 2 (FGFBP2), C—X3—C chemokine receptor 1 (CX3CR1), and GZMB]. Notably, the CD56 bright subset exhibits a more potent cytotoxic phenotype, characterized by the expression of multiple cytotoxic genes [175]. Subpopulation analysis has identified an NK cell subset with high expression of neural cell adhesion molecule (NCAM)1 and fc fragment of igg receptor III a (FCGR3A), as well as another subset characterized by high expression of keratins (KRT81 and KRT86), which may be enriched within tumor tissues [150]. Hu et al. identified a subset of NK cells that correlates with better clinical outcomes, suggesting a potential cytotoxic role in the TIME of ccRCC. However, the underlying mechanisms require further investigation [195]. Analysis using the scRNA-seq combined with VIPER algorithm has revealed heterogeneity in the spatial distribution of NK cell subsets. Specifically, NK cell subset 1 is relatively more abundant in adjacent normal tissues, while NK cell subset 2 is more prevalent in tumor tissues. Additionally, differential expression of certain proteins is observed, with some signature proteins exhibiting unique activity distribution patterns. This suggests a complex interplay between NK cell distribution and function within the TME [227]. In the single-cell data analysis of NK cells across various cancers, Tang et al. identified distinct compositions of tumor-infiltrating NK cells in RCC compared to other cancer types. Mature CD56 dimly expressed (dim)CD16 highly expressed (bright) NK cells were found to be more prevalent in RCC and exhibited unique distribution patterns. Extensive interactions with other cell types were observed, with the most notable being the strong interaction between CD56dimCD16brigh NK cells and lysosome—associated membrane protein (LAMP)3 + DCs, associated with the suppression of NK cell cytotoxicity [394]. Historically, NK cells were dichotomized into CD56dim and CD56bright populations based on CD56 expression levels, a classification that fails to elucidate the presence of extensive NK infiltration in renal cancers without successful tumor elimination. Liang et al. employed scRNA-seq to categorize NK cells in ccRCC into three distinct clusters: NK [early growth response gene (EGR)1], NK (GZMH), and NK [capping protein gelsolin – like** (**CAPG)]. These clusters show a stark contrast in prevalence within ccRCC compared to normal renal tissues, with the NK (EGR1) and NK (CAPG) subsets being disproportionately high in ccRCC. The NK (EGR1) subset is significantly correlated with metastatic spread, while the NK (CAPG) subset is linked to the activation of oncogenic pathways through FoxO and MAPK signaling. Moreover, NK (EGR1) is implicated in the metastasis of renal cancer to Hodgkin's lymphoma, and NK (CAPG) in its spread to T-cell leukemia, Ki-1 + anaplastic large cell lymphoma, and adult classic Hodgkin's lymphoma. Consequently, the NK (EGR1) and NK (CAPG) subsets are recognized as pivotal in the metastatic process of ccRCC [395].

NK cells exhibit extensive interactions with other cells within the TIME [396, 397]. ScRNA-seq analysis has identified NK cell populations expressing markers like NCAM1/CD56 and NCR1/NKp46. Within the CD8 + T cell population, a subset (CD8 TRM.3) exhibits high levels of cytotoxic genes [*GZMB*, prolactin—releasing factor 1*(PRF1)*] and markers for both CD8 + T cells (CD3E, CD8A) and NK cells [KLRB1, NCAM1/CD56, natural cytotoxicity triggering receptor (NCR)1, FCGR3A/CD16]. This suggests a mixture of NK cells and CD8 + T cells or features resembling NK cells, indicating a complex relationship between these cell types. The co-expression of markers may reflect cellular transformation, adaptive regulation in the TME, or a transitional state with mixed functions. This challenges traditional cellular classification and necessitates further investigation into the developmental, functional, and regulatory connections between NK cells and CD8 + T cells [225].

The potential of NK cells in cancer immunotherapy has been increasingly recognized [398–404]. In ICB therapy for ccRCC patients, scRNA-seq has identified gene expression differences related to NK cell function between responders and non-responders before treatment, though these differences are not significant. Certain NK cell-related gene expressions correlate with clinical response to nivolumab, with responders showing trends of change pre- and post-treatment. However, these changes are entangled with other immune cell gene expressions, complicating their direct attribution to clinical response. Further research is needed to clarify these relationships [226].

#### Dendritic cell

Dendritic cells (DCs) play a complex and multifaceted role in the TIME across various cancers, engaging in extensive interactions with other cellular components, thereby underscoring their significance in cancer progression [405–409]. DCs exhibit significant heterogeneity in terms of cell types and spatial distribution within the renal cancer TIME. ScRNA-seq analysis of ccRCC tumors and adjacent normal tissues has identified distinct subsets of DCs, including CD1C + myeloid DCs (mDCs). Furthermore, it has been observed that DCs engage in complex signaling with other immune cells, suggesting that the functional status of DCs may be associated with the immunomodulation of the tumor [328]. ScRNA-seq analysis of multi-regional tissues from renal cancer has identified three subsets of DCs: plasmacytoid DCs (pDCs), and cDC1 and cDC2, characterized by the expression of specific genes respectively. The proportions of these subsets, including cDC1 and cDC2, vary across different regions such as the tumor core, the tumor-normal tissue interface, and normal renal tissue [150, 329]. Among these subsets, the correlation between cDC1 cells and CD8 + T cells is particularly pronounced, suggesting that cDC1 may play a central role in activating and regulating CD8 + T cell-mediated anti-tumor immune responses [329]. Similarly, Mujal et al. discovered that distinct DC subsets play different roles in immune modulation. High infiltration of cDC1s is associated with effective immune responses of CD8 + T cells, highlighting their significance in antitumor immunity. In contrast, cDC2s are involved in immune regulatory processes related to CD4 + T cells, influencing the overall immune balance [240]. Phenotypic characterization of the TIME in ccRCC reveals that DCs express Fms—like tyrosine kinase** (**FLT)3, with DC1 cells additionally expressing CD141 and C - type lectin domain family 9 member A** (**CLEC9A). Furthermore, the migratory DC1 cluster exhibits enhanced expression of C - C chemokine receptor (CCR)7 [225].

DCs hold substantial promise in the field of tumor immunotherapy [410–415]. In the context of ICB therapy, scRNA-seq has identified two distinct clusters of DCs within the RCC TIME: CLEC9A + DCs and CD1c classical dendritic cells. The proportion of dendritic cells did not significantly change following ICB treatment in RCC patients, suggesting that they may not play a substantial role in ICB therapy. However, further research is required to validate this observation [226]. In contrast, another study conducted scRNA-seq on patients with ccRCC in the context of ICB therapy. This study observed changes in the expression of DC-related genes before and after treatment, suggesting a potential association between the expression of these genes and the response to ICB therapy. However, the study did not further elucidate the precise nature of this relationship or the underlying mechanisms involved [330]. Li et al. analyzed single-cell data from ccRCC and other cancers and revealed that ICB therapy reshapes the TIME. Responders exhibited anti-inflammatory and pro-inflammation traits in certain DC subsets, while non-responders showed inflammatory characteristics and increased IFN-α/γ responses in different states. These findings suggest that DC subsets may have differential roles in the response to ICB therapy [416].

#### Monocyte

Monocytes are considered precursor cells of macrophages and are an integral part of the TIME in many cancers [417–421]. Based on single-cell transcriptomic sequencing data from renal cancer, monocytes are categorized into distinct subsets. Specifically, circulating classical monocytes highly express CD14 but lack FCGR3A expression, whereas circulating non-classical monocytes exhibit high FCGR3A expression with low levels of CD14 [150, 225]. CD14 + monocytes are primarily found in the bloodstream, whereas CD14 + CD16 + monocytes exhibit characteristics of tissue residency [329]. Additionally, monocytes within the TME express S100A12 [288]. Borcherding et al. observed that the proportion of CD14 + monocytes is reduced, while the proportion of macrophages is increased in tissue-infiltrating myeloid cells compared to peripheral blood and normal kidney tissue [422]. Mujal et al. identified three discrete subsets of monocytes in the TIME of RCC. The first subset comprises CD14 + S100A8 + classical monocytes typically found in the initial phase. The second subset, termed"Mono-Int,"is characterized by CD14 positivity along with the expression of Ly6/PLAUR domain—containing proteins 3 *(LYPD3)* and *MHC-II* genes, reflecting a transitional state in their differentiation into macrophages. The third subset consists of FCGR3A + non-classical monocytes that exhibit expression of genes stimulated by interferon, akin to the"IFN-responsive"cells identified in murine tumor models [240].

Chen et al.'s research reveals that intercellular communication molecules, such as C3, EGFR, and HAVCR2, play a crucial role in the differentiation process of monocytes. The expression changes of these molecules are closely associated with the infiltration status of monocytes, indicating that the functional state of monocytes within the TME is intimately connected to cell-to-cell communication in ccRCC [301]. ScRNA-seq analysis of primary and metastatic CDRCC revealed that monocytes within the TIME interact with a subset of tumor cells through the frizzled class receptor (FZD)1/WNT5A ligand-receptor complex, participating in the regulation of osteoclast-associated processes. This suggests that monocytes contribute to the construction of the bone metastatic microenvironment in CDRCC [145].

#### B cell

B cells, as a major type of lymphocyte, play a complex role in the TIME, immunotherapy and targeted therapy, which is of great significance for cancer research [423–428]. In the analysis of single-cell transcriptome data from ccRCC, B cells are identified as one of the primary cellular components within the TIME. Although they constitute a relatively small proportion of the overall immune cell population and they are more abundant in adjacent normal tissues compared to tumor tissues [227], B cells in tumor tissues display distinct transcriptional profiles and maintain a stable proportion throughout disease progression. However, in advanced stages, the TIME may influence B cell functionality, leading to immunosuppressive characteristics. Thus, the specific functions and underlying mechanisms of B cells in ICB therapy require further investigation [225]. And the proportion of B cells present is associated with patient prognosis [256].Fitzsimons et al. conducted a comprehensive pan-cancer study of B cells using scRNA-seq, including RCC. They categorized RCC B cells into subgroups: naive, memory (classical and non-classical), activated, proliferating, and germinal center B cells. Plasma cells were divided into conventional, stress, immunoglobulin kappa constant (IGKC)-high, and metallothionein (MT)1X-high plasmablasts/plasma cells. Each subgroup has unique marker genes and functional states, and their differentiation trajectory was mapped. In RCC, B cell markers CD19 and membrane-spanning 4-domains subfamily A member 1 (MS4A1) initially increase and then decrease during differentiation [429]. Additionally, B cells are consistently found in renal cancer tissues, correlating with their numbers in adjacent non-neoplastic tissues. Characterization of B cell subsets identified tumor-associated atypical B cells (TAABs) with significant clonal expansion and proliferation, linked to activated CD4 + T cells. TAABs suggest a better prognosis but are rare in most renal cancer patients. Germinal center B cells (Bgc) in renal cancer exhibit dynamics similar to germinal center reactions in secondary lymphoid tissues. There is a shift in immunoglobulin classes from IgA to IgG in antibody-secreting cells, notably increasing IgG1 [430]. Further analysis using scRNA-seq data revealed specific splice isoform combinations in B cells, with different B cell subtypes showing variations. This suggests a correlation with subtype-specific B cell functions [345].

In the context of ICB therapy for renal cancer, scRNA-seq and other technologies have revealed a significant presence of B cells at baseline in responders compared to non-responders. However, this difference was not observed during the course of treatment. Notably, while there was a trend towards increased B cell numbers and GZMB expression in responders, these changes did not reach statistical significance in bulk RNA sequencing. This suggests that B cells may play a complex role in ICB therapy and further investigation is needed to understand their specific functions and mechanisms of action [330].

In terms of targeted therapy, scRNA-seq analysis of ccRCC has revealed that genes associated with sorafenib resistance are significantly enriched in the marker genes of immune cells, such as macrophages, B cells, and T cells, while sensitive genes are enriched in the marker genes of MCs [156]. Analysis of tumor tissues from ccRCC patients receiving pazopanib as neoadjuvant therapy showed changes in the proportions of MCs, ECs, and T cells before and after treatment. Non-responsive patients displayed reduced levels of T cell exhaustion and increased senescence in both T and B cell populations [431].

These insights enhance our comprehension of the role and composition of B cells within the renal cancer TME and may inform the development of targeted immunotherapies, potentially leading to more personalized treatment strategies.

#### Mast cell

Although mast cells have been less studied in the TIME, they are still an important component of the TIME [432–436]. Research on them is equally important for revealing the overall landscape of the TIME. Cheng et al. conducted a single-cell analysis of myeloid cells across various cancers. Intriguingly, they found that mast cells are relatively more abundant in renal cancer compared to other cancer types. Moreover, the frequency of VEGFA + mast cells is higher than that of TNF + cells, suggesting their role in promoting angiogenesis in renal cancer [437]. ScRNA-seq and scATAC-seq data analyses have identified mast cells in ccRCC, characterized by the expression of marker genes kinase insert domain receptor (*KIT*) and tryptase alpha/beta 1(*TPSAB1*) [288]. Additionally, single-cell analysis of various RCC subtypes has revealed a higher proportion of mast cells in type 1 pRCC compared to type 2 pRCC and ccRCC [438]. Furthermore, Hu et al. observed that mast cells in the TIME of ccRCC are associated with better prognosis, suggesting a potential immunological function in tumor resistance [195].

#### Neutrophil

**Fig. 4:**
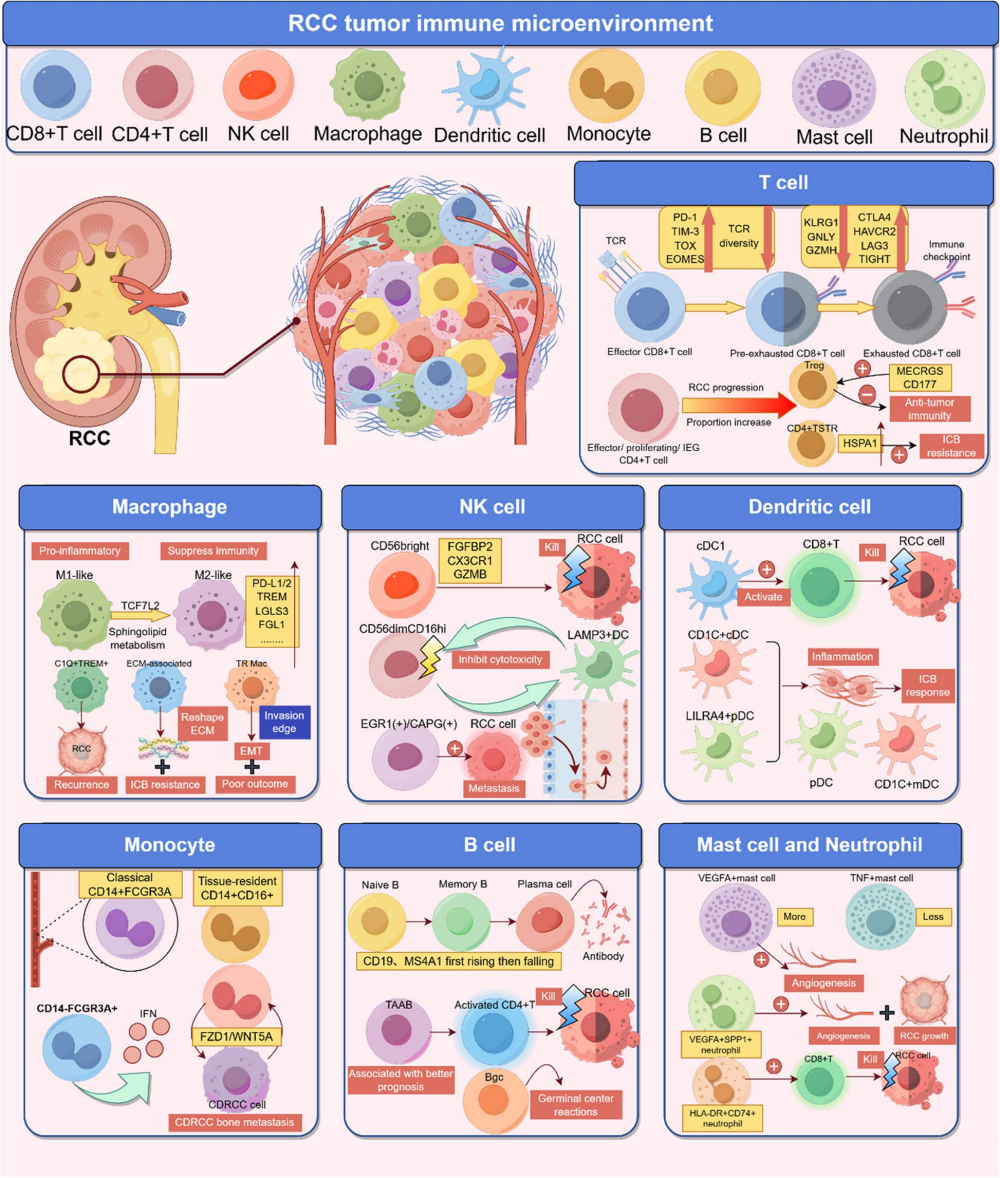
The intricate panorama of RCC TIME. In the TME of RCC, immune cells significantly outnumber tumor cells. Mapping the immune landscape of RCC is essential for deepening our understanding of the disease and for the development of therapeutic strategies. The immune microenvironment of RCC encompasses a variety of immune cells, with CD8+ T cells predominating, followed by NK cells, dendritic cells, monocytes, B cells, mast cells, and neutrophils. These immune cells do not exist in isolation but engage in complex interactions with other immune and tumor cells, creating an immunological niche that supports the growth and metastasis of RCC. This figure was created based on the tools provided by Figuredraw.com

Neutrophils are an integral part of the TIME and play a multifaceted role in various cancers and immunotherapy [439–445]. However, there is a need for further research on their involvement in the TIME. Wu et al. conducted a single-cell transcriptomic analysis of neutrophils across different cancers and identified heterogeneity in renal cancer. They discovered two subgroups: VEGFA + SPP1 +, which is significantly overexpressed in renal cancer and linked to tumor angiogenesis and growth, and HLA-DR + CD74 +, which is present in renal cancer and contributes to antitumor immunity by activating T cells. The proportion of the HLA-DR + CD74 + subgroup is associated with better patient prognosis [446]. In the analysis of myeloid cells, researchers observed that neutrophils are significantly less abundant in both the PT and TT compared to the ART. This differential distribution suggests that tumor cells may evade the clearance by circulating neutrophils through certain mechanisms, thereby establishing an immunosuppressive state within the TME that is conducive to tumor growth and progression [176]. Analysis of scRNA-seq data from RCC has revealed a significant correlation between neutrophils and macrophages, suggesting that neutrophils may differentiate into macrophages. Furthermore, they communicate with other cells through pathways such as the adhesion G protein—coupled receptor (ADGR)E5 signaling, participating in tumor-associated biological processes that impact the initiation and progression of cancer [447]. The panorama of RCC TIME is summarized in Fig. 4.

### Single-cell sequencing technology provides a detailed view of the tumor matrix microenvironment in renal cancer

#### Endothelial Cell

ECs are a crucial component of the TME and play a significant role in tumor angiogenesis [448–452]. In renal cancer, the proportion of ECs varies and is associated with tumor stage and patient prognosis [256]. In the context of renal cancer, ECs within the TME exhibit heterogeneity. ScRNA-seq analysis has revealed distinct subpopulations of ECs in renal cancer. For example, IGF—binding protein (IGFBP)3 + ECs and collagen-producing ECs, exhibiting spatial heterogeneity within the tumor. IGFBP3 + ECs are enriched in tumor tissues, whereas collagen-producing ECs are more prevalent at the tumor-normal tissue interface, potentially contributing to the ECM [150]. Additionally, Young et al. determined that tumor-associated endothelial cells (TECs) in RCC primarily derive from the ascending vasa recta and classified them into two subsets, tE1 and tE2. Receptors of the VEGF signaling pathway, such as kinase insert domain receptor (KDR), FLT1, and FLT4, are mainly expressed in tE1, while tE2 expresses VEGFC and FLT1, relevant to lymphangiogenesis. Additionally, tE2 ECs highly express atypical chemokine receptor (ACKR)1, aiding immune cell migration, thereby illustrating the complex VEGF signaling network within RCC tissue [121]. Hu et al. identified six subgroups of ECs in ccRCC. Three subgroups [serine proteinase inhibitor clade E member 2(SERPINE2) +, CCL23 +, and sclerostin (SOST) +] mainly originate from normal tissue, while subgroups 6 (ACKR1 +) and 1 [potassium voltage—gated channel subfamily E regulatory subunit 3 (KCNE3) +] are enriched in tumor tissue. Ectonucleotide pyrophosphatase (ENPP)2 and von willebrand factor (VWF) are potential markers for tumor-derived ECs. Subgroup 1 is characterized by suppressed inflammation and enrichment of WNT-β-catenin, hedgehog, and NOTCH signaling pathways. Conversely, subgroup 6 shows high proliferation and inflammation, linked to poor prognosis. Both subgroups 1 and 6 are enriched in KRAS signaling and angiogenesis pathways [195]. Compared to benign adjacent renal tissue, which contains five endothelial subpopulations, ccRCC tumors mainly consist of ascending vasa recta (AVR)−1 type ECs. The AVR-1 subset shows elevated VEGF receptor mRNA levels and upregulates genes like endothelin receptor type (*EDNR)B*, *VWF*, and heparan sulfate proteoglycan (*HSPG)2*, while downregulating the IFN-γ response and upregulating the EMT pathway. In primary ccRCC patients, a higher EC fraction correlates with better overall survival. However, in metastatic ccRCC patients undergoing immunotherapy, a higher EC fraction is linked to non-response to treatment [122]. Heterogeneity among ECs has also been observed in different grades of ccRCC. Chromatin—remodeling binding protein (CHRBP)3 + capillary ECs, enriched in LG tumors and associated with better overall survival, while venous ECs, enriched in HG tumors, show no significant prognostic differences based on their gene signature. Arterial and capillary ECs share the differential gene *ENPP2*, and high *ENPP2* expression is linked to better prognoses, suggesting its potential as a biomarker and prognostic indicator. Conversely, high expression of certain metabolism-related genes in venous ECs is associated with poorer outcomes [207]. J. Li et al., using pan-cancer scRNA-seq, identified various endothelial subpopulations, finding hypoxia-associated ECs enriched in RCC. Venous subgroups E09 and E10 in RCC had higher collagen-related gene expression and angiogenic potential, indicating their role in vascular and matrix remodeling. In contrast, subgroup E06 showed lower collagen gene expression and angiogenic scores but had strong leukocyte-endothelial interactions and T cell association. Spatial analysis of RCC tissues revealed decreased capillary density and increased tip cell abundance from non-neoplastic to tumor regions, underscoring the importance of vascular architecture changes in RCC progression [453]. Within the RCC TME, TECs engage in a variety of interactions with other cellular components. Specific interactions, such as CD99-PILRα, suggest a role in reducing immune infiltration, while LGALS9-HAVCR2/TIM-3 interactions indicate an immunosuppressive function of ECs. These findings reveal the immunomodulatory functions of ECs within the TME [130]. Additionally, a subset of ECs in ccRCC has been found to express fibroblast markers, including periostin (POSTN) and COL3A1, suggesting a potential association with fibroblasts [454]. The spatial distribution of ECs within ccRCC and chrRCC tumors has also been investigated. In particular, an increase in the number of ECs at the tumor periphery in a CN may be associated with tumor cell invasion and proliferation, highlighting the spatial heterogeneity of EC distribution [123]. Gene expression studies have shown that ECs in RCC exhibit heterogeneity and differential expression of genes through alternative mRNA splicing. Differentially regulated genes and differential mutations have been identified among ECs, indicating specific genetic alterations within the TME [345]. Different subpopulations of ECs have been characterized based on their gene expression profiles, including those associated with tumor angiogenesis, renal cancer cell proliferation and metastasis, glomerular integrity, hematopoietic stem cell support, and high angiogenesis scores [175]. Beyond the aforementioned genes, certain EC subpopulations also highly express genes such as platelet—endothelial cell adhesion molecule (*PECAM)1*, cadherin (*CDH)5*, and *VWF*. Interestingly, one subset not only exhibits endothelial characteristics but also expresses markers typical of CAFs, including TAGLN, actin alpha 2 smooth muscle aorta (ACTA2), COL1A2, and platelet—derived growth factor receptor beta (PDGFRB). This phenomenon suggests that in the TIME of ccRCC, there may be complex interplays or transitions between ECs and fibroblasts, potentially play a role in processes such as tumor progression, angiogenesis, and ECM remodeling [255]. Integrating scRNA-seq and scATAC-seq identified two distinct EC subpopulations within ccRCC. One subpopulation expresses VCAM1, while the other lacks VCAM1 but is enriched for KDR. These subpopulations display significant gene expression differences: VCAM1-negative cells predominantly express genes linked to endothelial proliferation and vascular development, whereas VCAM1-positive cells highly express genes regulating immune cell chemotaxis and migration [288]. Shi et al. classified ECs from PT and TT into six subgroups based on gene expression: glomeruloid ECs (Endo1: *SOST* +), cancer-associated ECs (Endo2: *NDUFA4L2* +), arterial ECs [Endo3: gap junction protein alpha 5 (*GJA5)* +], ACKR1 + ECs (Endo4: *ACKR1* +), tip cells (Endo5), and *CXCR4* + cells (Endo6). Endo2 is more prevalent in TT than in ART and PT, with TT ECs showing increased ECM remodeling and cell proliferation, potentially aiding TT growth. In contrast, PT ECs have altered interferon response and antigen binding, with some subsets being more immunosuppressive. The gene signature of Endo2 correlates with poor prognosis, indicating its potential as a prognostic biomarker [176]. Zvirblyte et al. identified five endothelial subpopulations in ccRCC, with the Tumor vasculature 3 subgroup displaying a unique tip cell phenotype, expressing genes like lymphocyte antigen 6 complex locus H (*LY6H)*, placental growth factor (*PGF)*, lysyl oxidase (*LOX)*, carbohydrate sulfotransferase 1(*CHST1)*, and type IV collagen. Overexpression of EMT-related genes in this subgroup and the tumor AVR-like vascular subgroup correlates with poorer overall survival. TECs modify the ECM by depositing various components and expressing modifiers [LOX and peroxidasin (PXDN)], actively participating in immune suppression and angiogenesis within the TME. They interact with immune cells, transmitting suppressive signals via TIGIT-NECTIN2 and HLA-F-LILRB1/2 pathways and promoting endothelial migration and proliferation through TNF-NOTCH1 interactions [177]. To further elucidate the role of tip cells in RCC, an integration of scRNA-seq data from cancerous stromal cells across various malignancies revealed that PGF + tip cells are relatively enriched in RCC and correlate with poor prognosis. Moreover, their enrichment is significantly associated with EMT scores, suggesting their involvement in the invasion and metastasis of RCC cells. Notably, PGF + tip cells are highly enriched in patients with an immuno-exhausted TME, characterized by a marked reduction in lymphocytic infiltration [455]. The integration of snRNA-seq with multi-omics technologies has advanced our understanding of ECs in ccRCC. Within the IM1 and IM2 subtypes of the tumor, ECs exhibit elevated expression of *FOS*, *JUND*, and *FOSB* genes, which are known to activate endothelial cell proliferation. Furthermore, snATAC-seq data confirmed increased chromatin accessibility for these genes [190]. In the analysis of cellular signaling pathways, it has been observed that vascular EC signaling is more pronounced in ccRCC compared to pRCC. This differential signaling profile indicates a potential reason why ccRCC exhibits a better response to anti-angiogenic therapy compared to pRCC [438]. In the study of the tumor matrix microenvironment of nccRCC, ECs are categorized into subsets: tumor-derived ECs marked by secreted protein acidic and rich in cysteine – like (SPARCL)1 and normal-derived ECs marked by corticotropin—releasing hormone—binding protein (CRHBP). SPARCL1, a matrix cell protein, enhances tumor neovascularization, invasion characteristics, and modulates endothelial cell heterogeneity based on the TME. Normal ECs tend to evolve into TECs, and under TME influence, ECs upregulate hypoxia and angiogenesis pathways [213].

#### Fibroblast

Fibroblasts, as a significant component of the tumor stroma, have been increasingly recognized for their complex and critical roles in cancers [456–462]. Moreover, it often shows heterogeneity in various cancers and constitutes a complex ECM microenvironment [463–466]. In ccRCC, fibroblasts have been found to be more abundant compared to adjacent normal tissue [227]. Fibroblasts in ccRCC can be categorized into subtypes based on their gene expression profiles. For example, Hu et al. discovered that the majority of fibroblasts originate from tumor tissue, and interestingly, they almost uniformly express the myofibroblast marker α- smooth muscle actin (SMA). These cells can be categorized into three subtypes: subgroup 1 [CEBPB + PDGFRB + fatty-acid-binding protein (FABP)5 +], subgroup 2 (lacking specific markers), and subgroup 3 (IL32 + NNMT + COL1A1 +). FABP5 is almost exclusively expressed in tumor-derived fibroblasts, and CEBPB is associated with lipid metabolism, indicating aberrant lipid metabolism in CAFs within ccRCC [195]. Li et al. identified a subset of collagen-expressing fibroblasts that are enriched at the tumor-normal tissue interface. This subset co-localizes with other ECM-producing stromal cells and may function in the remodeling of the extracellular environment and in cell–cell interactions [150]. Furthermore, CAFs in ccRCC can be categorized into distinct subclasses, including inflammatory (iCAFs), antigen-presenting (apCAFs), and myofibroblast (myCAFs). MyCAFs express genes associated with TGFβ activation and myofibroblast markers, potentially influencing the regulation of immune cells and the remodeling of the tumor stroma within the TME [131]. And certain TFs are significantly enriched in i-CAFs, including EGR1, JUND, X—box—binding protein** (**XBP)1, activating transcription factor** (**ATF)3, and JUNB [467]. Additionally, fibroblasts can also be further categorized into"immunoregulatory CAFs"and"TEX-related CAFs,"with the latter associated with T cell exhaustion and promoting tumor cell proliferation, invasion, and migration through the SLC38A5-CCL5 axis [468]. Fibroblasts in RCC also exhibit gene expression heterogeneity. Similar to ECs, scNanoGPS analysis revealed 137 DCI genes in fibroblasts. The expression patterns of these isoforms are closely associated with the functions of fibroblasts within the TME, including their involvement in processes such as extracellular matrix remodeling [345]. CAFs in ccRCC express signature genes such as fibroblast activation protein *(FAP)*, leucine—rich repeat—containing *(LRRC)15*, fibronectin *(FN)1*, transforming growth factor beta induced *(TGFBI)*, and thyroid—stimulating hormone *(THY)1*, and it has been observed that these expressions correlate with patient outcomes [175]. Fibroblasts are more abundant in LG tumors but relatively scarce in HG tumors [207]. In recurrent RCC (reRCC), single-cell transcriptomic studies have identified an increase in CAFs and a reduction in CD8 + T cells within reRCC tumors. Notably, CAFs highly express LGALS1, which induces apoptosis in CD8 + T cells, leading to immunosuppression and suggesting a significant role for CAFs in RCC recurrence [469]. In terms of immunotherapy, high expression of CXCL14 has been associated with improved overall survival after immunotherapy in RCC patients. ScRNA-seq analysis has identified a CXCL14 + fibroblast cluster in immunotherapy-responsive RCC patients, suggesting that fibroblasts may influence mechanisms of immunotherapy response [470].

Among RCC subtypes, CAFs show subtype-specific heterogeneity. Using scRNA-seq, Xu et al. identified seven CAF subgroups with unique transcriptional signatures, variably present in ccRCC, chrRCC, and pRCC. At the pathway level, CAFs exhibit significant metabolic and regulatory diversity. In ccRCC, CAFs and macrophages engage in bidirectional signaling, absent in chrRCC. Furthermore, fibroblasts in chrRCC lack interaction with other cell types [471]. In studies of Wilms tumor, a cluster composed of Wilms MCs and non-neoplastic ccRCC fibroblasts has been identified, exhibiting a fibroblast-to-myofibroblast transcriptional signature [121]. scRNA-seq of Wilms tumor organoids has identified the presence of cell populations resembling fibroblasts. These cells express a variety of fibroblast-associated markers, including collagens, and form a complex cellular network within the organoids, contributing to the heterogeneity of Wilms tumor organoids [472]. In RMC, two CAF subtypes have been identified: myCAF and iCAF. MyCAF is mainly found in post-treatment tumors, while iCAF predominates in untreated PT and is present in both primary and metastatic tumors. MyCAF is partially found in PT but underexpressed in metastatic tumors. Renal CAFs may derive from renal mesenchymal cells, contributing to the formation of the RMC TME [144]. In FHRCC, scRNA-seq has identified fibroblasts marked by phospholipase A2 group IIA (PLA2G2A) and CXCL14 expression, particularly a fibroblast subtype with high expression of PLA2G2A and CXCL14 found in patients with PR. This subtype has previously been reported to be associated with poor prognosis in pancreatic ductal adenocarcinoma, but in FHRCC, it may indicate a positive response to immunotherapy. The specific mechanisms underlying this phenomenon require further investigation [242]. In nccRCC, fibroblast have attracted significant attention. Using scRNA-seq on tumors and normal tissues from various nccRCC subtypes (pRCC, chrRCC, CDRCC, and sarRCC), fibroblasts were categorized into four subgroups with distinct gene expression profiles. The fetal cluster expresses Polo—like kinase (PLK)2, cluster 1 expresses NDUFA4L2, aiding cancer cells via OXPHOS, cluster 2 expresses S100A8, linked to immune modulation, and cluster 3 expresses THBS2, with TFs CCAAT—enhancer—binding protein delta (CEBPD) and FOXO3 upregulated, associated with adipocyte differentiation and tumor promotion. Additionally, cluster 1 shows HIF1α and PPARγ upregulation, linked to lipid accumulation [213].

CAFs in RCC TME also participate in various crosstalk with other cells. For example, fibroblasts in ccRCC accumulate in the IM4 subtype, which is attributed to the enrichment of IGF1 + macrophages that promote their survival and migration [190]. In renal cancer bone metastasis samples, fibroblasts exhibit increased expression of COL3A1 and EMP1, indicating their involvement in bone metastasis [162].

In terms of myofibroblasts, Shi et al. classified myofibroblasts into five subgroups: Myo1 (high heat shock protein expression, indicating stress), Myo2 (highly perivascular phenotype), Myo3 [actin gamma (ACTG)2 + fibroblast-associated], Myo4 (antigen-presenting phenotype), and Myo5 (CAF features). The perivascular-like Myo2 subgroup is more prevalent in PT and TT than in ART, possibly due to its role in tumor vasculature maturation and remodeling. Myofibroblasts in PT are enriched in TNFα response pathways, while those in TT are active in angiogenesis and ECM remodeling pathways, including EMT, collagen fiber organization, and ECM organization. In sunitinib-treated patients, higher Myo2 signature scores correlate with longer PFS, indicating Myo2 as a potential predictor of TKI response in ccRCC patients [176]. Additionally, Zhou et al. identified that myofibroblasts in ccRCC exhibit robust activity and functionality within the TME, with high scores for the stemness gene *CD44*. They are enriched in OXPHOS and related pathways, and were categorized into subgroups C0, C2 (FXYD2 +), C3 [high—mobility group A (HMGA)1 +], C4 (ITGA1 +), and C5 [pituitary tumor—transforming gene (PTTG)1 +]. Notably, the C3 subgroup is abundant and pure in tumor tissues, has a large proportion in the S phase, and demonstrates strong proliferative capacity. These cells interact intensively with MCs through the MPZ signaling network and have high gene scores for MPZ like-1 (*MPZL1)*. A prognostic model based on related genes is effective, and *MPZL1* promotes the proliferation, migration, and invasion of ccRCC [199]. Furthermore, Zvirblyte et al. identified that myofibroblasts in the TME of ccRCC express abundant ECM components, such as collagen types I, III, IV, and VI, as well as fibronectin, with enrichment of related markers like ACTA2 and tissue inhibitor of metalloproteinase (TIMP)1. Moreover, myofibroblasts are closely associated with immune cells. For instance, they interact with CTLs through the HLA-E-KLRC1 axis. This communication feature is associated with increased gene expression as ccRCC progresses [177].

#### Pericyte

Pericytes, which surround microvessels and play a crucial role in vascular stability and function [473–477], contribute to the tumor matrix microenvironment in various cancers [478–482] and exhibit significant heterogeneity within them. In ccRCC, scRNA-seq analysis has identified two distinct pericyte subpopulations. Pericyte-1 exhibits a pro-inflammatory angiogenic phenotype, characterized by high expression of genes associated with angiogenesis and inflammation (such as *RGS5*, *THY1*, *AXL*, *KCNJ8*), indicating a role in promoting tumor angiogenesis. Conversely, Pericyte-2 displays a vSMC phenotype, expressing genes [*ACTA2*, phospholamban (*PLN)*, *TAGLN*, ras—related and estrogen—regulated growth inhibitor–like (*RERGL*)] essential for vascular structural integrity. The decline in Pericyte-2 as ccRCC progresses indicates a tumor-induced vascular remodeling shift favoring pericytes with pro-inflammatory and pro-invasive traits [175]. A subset of pericytes expressing RGS5 can be categorized into subpopulations linked to classical mesangial cells and those involved in CAF formation. Trajectory analysis indicates that pericytes may transition into other cell types through pathways like transforming into myCAFs via the TGFβ-driven pathway or into inflammatory state of mesangial cells (MSG.inf cells), which express stress and inflammation-related factors, through the IL1a response pathway [131]. Additionally, scRNA-seq analysis has identified pericytes as a key source of PGF in RCC. These pericyte-derived factors, including PGF, interact with MCs and ECs within the TME, promoting angiogenesis. The pro-angiogenic factor network in RCC is enhanced by PGF from pericytes and VEGF from tumor and stromal cells, facilitating nutrient supply to the tumor and promoting metastasis [483]. Moreover, a comprehensive analysis of scRNA-seq data from diverse cancer stroma identified distinct pericyte subsets within RCC. Specifically, fibrogenic and angiogenic pericytes were linked to unfavorable outcomes. These subsets were found to interact intensively with PGF + tip cells, potentially contributing to ECM accumulation through interactions involving laminin, collagen, ITGA, and ITGB, and to vascular dysfunction through VEGFA-VEGFR and WNT5A- melanoma cell adhesion molecule (MCAM) pathways, which may promote RCC progression [455].

These findings highlight the heterogeneity and complex roles of pericytes in ccRCC, including their involvement in tumor angiogenesis, vascular remodeling, and interaction with other cellular components within the TME.

#### Other stromal cell

**Fig. 5:**
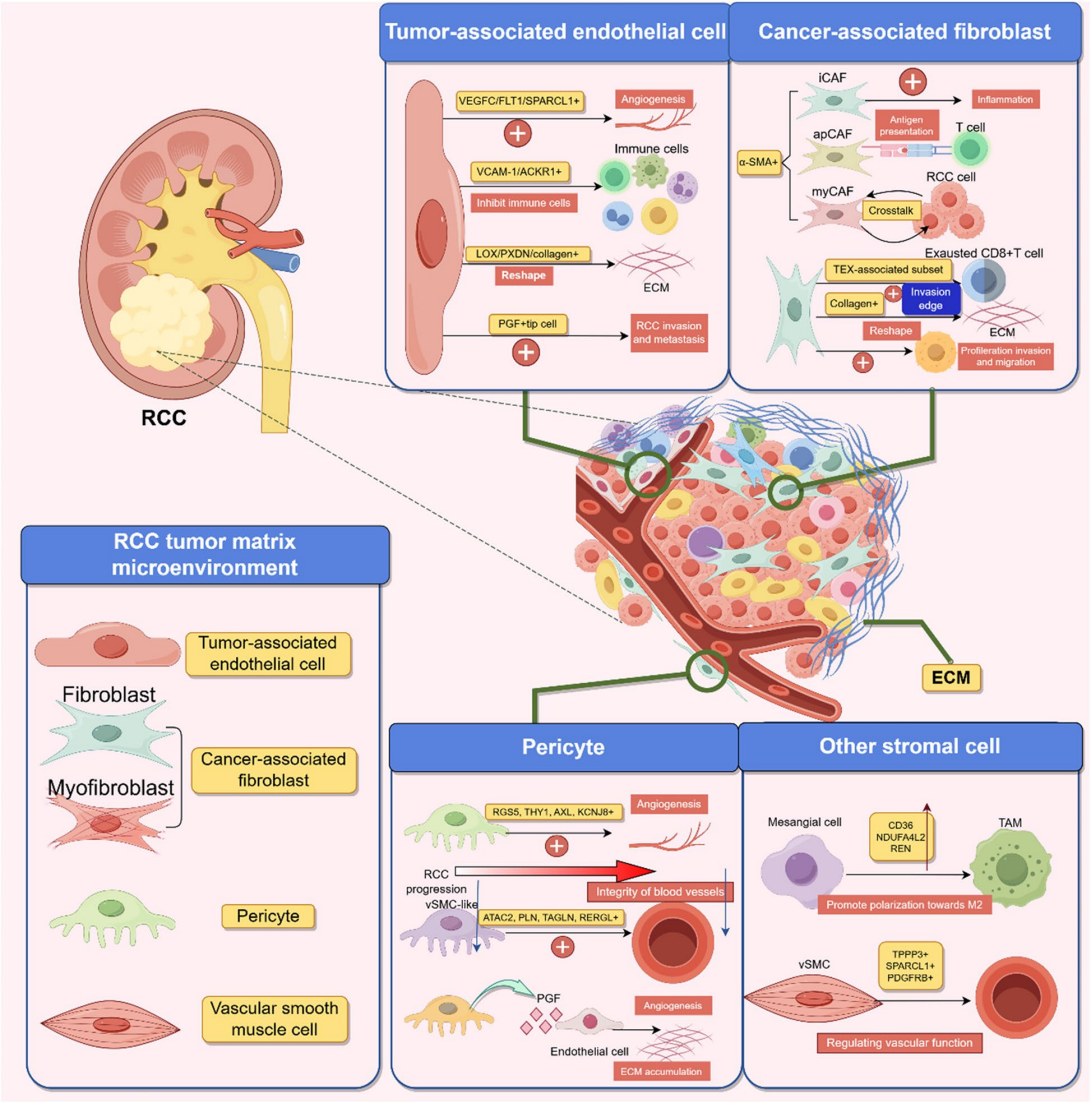
The landscape of RCC tumor matrix environment. In the TME of RCC, stromal cells constitute a significant component, interacting with the immune microenvironment and tumor cells to form the TME. The matrix microenvironment includes a variety of cell types such as tumor-associated endothelial cells (TECs), cancer-associated fibroblasts (CAFs), pericytes, and vascular smooth muscle cells (vSMCs). These cells not only interact with each other but also have extensive connections with tumor cells, playing crucial roles in tumor angiogenesis, EMT, invasion, and metastasis in RCC. This figure was created based on the tools provided by Figuredraw.com

Mesangial cells, a specialized cell type located within the glomerulus, play a crucial role in the structure and function of the glomerulus, and their dysfunction is associated with the onset and progression of various kidney diseases [484]. Mesangial cells exhibit heterogeneity within the renal cancer stroma. Through scRNA-seq and spatial genomics techniques, distinct subpopulations of mesangial cells have been identified, including smooth muscle cells (SMCs), classical mesangial cells (MSG), and MSG.inf. MSG expresses PDGFRB and RGS5, SMCs express smooth muscle markers, and MSG.inf characteristically expresses factors related to stress and inflammation [131]. In ccRCC, Zvirblyte et al. used scRNA-seq to reveal complex marker gene expression patterns in mesangial cells. They observed upregulation of vSMC and mesangial cell markers like PDGFRB, along with significant transcriptional differences between tumor and healthy tissues. Mesangial cells in tumors highly express genes such as *CD36* (linked to chronic kidney disease), *NDUFA4L2*, and renin (*REN*), indicating a reactive nature to the TME. These cells also interact with immune cells, primarily directing immune-suppressive signals to TAM subsets 1 and 2. For example, the annexin (ANX)A1-formyl peptide receptor (FPR)1 interaction is involved in anti-inflammatory macrophage polarization and tumor progression. Additionally, interactions with CTLs via HLA-E-KLRC1 correlate with poorer patient survival, with increased expression as the disease progresses [177]. In CDRCC, analysis of scRNA-seq has identified vSMCs as a distinct cluster within cellular populations, characterized by the specific expression of genes such as tubulin polymerization promoting protein family member 3 (*TPPP3*), *SPARCL1*, and *PDGFRB* [145]. These vSMCs are hypothesized to play a crucial role in maintaining microenvironmental stability and regulating the function of tumor vasculature. However, the precise mechanisms underlying their interaction with MCs require further investigation. Understanding these mechanisms is significant for elucidating the pathogenesis of CDRCC and for identifying potential therapeutic targets. The landscape of RCC tumor matrix microenvironment is summarized in Fig. 5 and the panorama of RCC TME is summarized in Table 1.

**Table 1:** The panorama of the RCC TME, including TIME and matrix microenvironment. The TME of RCC comprises a variety of cell types, including immune cells, stromal cells, and tumor cells. These cells do not exist in isolation but engage in extensive interactions and connections, collectively forming a microenvironment conducive to the growth and progression of RCC. Single-cell resolution analysis provides a clearer and more precise understanding of the RCC TME, facilitating research on RCC and contributing to clinical treatment strategies

Cell type	Functions in the RCC TME	Reference
Immune cells		
CD8 + T cells	During the progression of RCC, CD8 + T cells undergo a transition from a functional to an exhausted state, characterized by a reduction in TCR diversity, decreased expression of cytotoxic genes, and an increasing expression of immune checkpoint molecules. Their metabolic activity initially increases and then declines, indicating a shift from an active to a dysfunctional state. The interaction of CD8 + T cells with tumor cells, TAMs, and Tregs contributes to this exhaustion process. The loss of CD8 + T cell function is associated with higher tumor grades, advanced pathological staging, and distant metastasis. Furthermore, responders and non-responders to ICB therapy exhibit distinct dynamics, heterogeneity in CD8 + T cell subsets, clonal expansion of TCRs, and gene expression profiles, which ICB therapy reshapes in RCC patients'TME. This reshaping provides insights into the mechanisms of response to immunotherapy and resistance to ICB treatment. CD8 + T cells display significant heterogeneity within the TME, which can be categorized into various subsets based on gene expression and molecular markers, each with distinct functional states. This heterogeneity offers clues for further understanding the evolution of RCC	Yang et al. [207] Braun et al. [225] Li et al. [150] Mujal et al. [240] Alchahin et al. [175] Bi et al. [226] Long et al. [288]
CD4 + T cells	CD4 + T cells exhibit significant heterogeneity at the single-cell resolution within the TME of RCC. Distinct subsets of these cells possess varying functional states and gene expression profiles. Notably, Tregs are highly enriched in RCC and mediate immune suppression and resistance to immunotherapy through their interactions with other immune cells, tumor cells, and stromal cells	Krishna et al. [329] Kinget et al. [328] Hu et al. [195] Chevrier et al. [304]
Macrophages	Macrophages within the TME of RCC exhibit significant heterogeneity. At a single-cell resolution, they can be finely classified, surpassing the traditional M1/M2 classification method. In RCC immunotherapy, pro-inflammatory (M1-like) macrophages are beneficial to treatment, while anti-inflammatory (M2-like) macrophages are detrimental. TREM + C1Q + macrophages have been identified as closely associated with high recurrence risk, poor prognosis, and resistance to ICB treatment in RCC, suggesting their potential as biomarkers for predicting recurrence and prognosis. Additionally, macrophages participate in the remodeling of the ECM, thereby promoting RCC metastasis	Bi et al. [226] Aggen et al. [365] Obradovic et al. [227] Alchahin et al. [175] Litchfield et al. [382] Shi et al. [176]
NK cells	Natural killer (NK) cells within the TME of RCC are divided into distinct subsets, such as CD56 +, CD56bright, and CD56dim, indicating their heterogeneity. These subsets engage in complex intercellular communications with other cells. Notably, LAMP3 + DCs interact significantly with the CD56dimCD16hi NK cell subset, suppressing the anticancer toxicity of NK cells. Additionally, the NK (EGR1 +) and NK (CAPG +) subsets are considered key cells in the metastatic process of RCC	Li et al. [150] Tang et al. [394] Liang et al. [395]
Dendritic cells	Dendritic cells (DCs) within the TME of RCC exhibit subset and spatial distribution heterogeneity, including plasmacytoid DCs (pDCs) and conventional DCs (cDCs). Among these, cDC1 plays a central role in mediating T cell immunity. Furthermore, during immunotherapy, DCs in RCC display various states, ranging from pro-inflammatory to anti-inflammatory characteristics. There is a notable difference in the functional states of DCs between non-responders and responders to immunotherapy, suggesting their potential role in the response to treatment	Li et al. [150] Krishna et al. [329] Li et al. [416]
Monocytes	In the TME of RCC, monocytes are categorized into distinct subpopulations, such as CD14 + FCGR3A-, CD14-FCGR3A +, and CD14 + CD16 + monocytes, demonstrating their heterogeneity. The complex intercellular communications within the TME influence the differentiation of monocytes, and these cells participate in the osteotropic metastatic process of CDRCC	Krishna et al. [329] Braun et al. [225] Pan et al. [145] Chen et al. [301]
B cells	B cells within the TME of RCC are present in relatively low proportions but exhibit stable ratios. Their differentiation trajectory initiates from naive B cells, progresses through memory B cells, and ultimately terminates at plasma cells. Among the B cell subsets, tumor-associated atypical B cells (TAABs) are enriched in RCC and are associated with better prognosis for RCC patients. Furthermore, certain sorafenib resistance genes are enriched in B cells, suggesting their involvement in resistance to targeted therapies. The presence of TAABs and their interaction with other immune cells, including CD4 T cells, highlight their potential as prognostic markers and their role in the TME	Braun et al. [225] Obradovic et al. [227] Fitzsimons et al. [429] Yang et al. [430] Zhang et al. [156]
Mast cells	Mast cells in the TME of RCC are more abundant compared to other cancers, with a higher frequency of VEGFA + mast cells than TNF + cells, suggesting their role in promoting cancer angiogenesis. Additionally, mast cell abundance is higher in type 1 pRCC compared to ccRCC and type 2 pRCC. In terms of prognosis, mast cells may correlate with better outcomes for ccRCC patients	Cheng et al. [437] Young et al. [438] Hu et al. [195]
Neutrophils	Neutrophils in the TME of RCC exhibit heterogeneity, dividing into two subsets, including VEGFA + SPP1 + neutrophils, which are significantly overexpressed in RCC and associated with tumor angiogenesis and growth; and HLA-DR + CD74 + neutrophils, which activate T cells to promote antitumor immunity. A reduction in neutrophils has been observed in tumor thrombi (TT) of RCC, suggesting that RCC may suppress the immune surveillance function of neutrophils through certain pathways, thereby acquiring the ability to invade and metastasize	Wu et al. [446] Shi et al. [176]
Stromal cells		
Endothelial cells	Within RCC, endothelial cells comprise multiple subsets, each expressing distinct molecules and fulfilling unique functions. These include participating in the remodeling of the RCC, engaging in the VEGF regulatory network, facilitating vascular restructuring and tumor angiogenesis, and suppressing the antitumor activities of immune cells. Furthermore, certain subsets of endothelial cells are indicative of prognosis and responsiveness to immunotherapy	Li et al. [150] Young et al. [121] Li et al. [453] Zhang et al. [130] Zhang et al. [122]
Fibroblasts	In RCC, the majority of fibroblasts originate from tumor tissues and exhibit significant heterogeneity. Cancer-associated fibroblasts (CAFs) are categorized into various subclasses, including inflammatory (iCAF), antigen-presenting (apCAF), myofibroblast (myCAF), immunomodulatory CAF (immuno-CAF), and TEX-associated CAF, among others. These CAFs interact with other cells within the TME and participate in processes such as ECM remodeling, RCC tumor invasion and migration, and suppression of immune cell anti-tumor activity. Additionally, certain subsets of CAFs can serve as potential predictive markers for prognosis, response to immunotherapy, and targeted therapy, as well as recurrence	Li et al. [150] Davidson et al. [131] Chen et al. [468] Du et al. [162] Shi et al. [176] Pan et al. [470]
Pericytes	Pericytes in RCC can be categorized into two distinct subpopulations, each with unique molecular signatures and functional roles. One subset exhibits a pro-inflammatory angiogenic phenotype, characterized by the high expression of genes related to angiogenesis and inflammation, suggesting their role in promoting tumor angiogenesis. The other subset presents a vascular smooth muscle cell (vSMC) phenotype, expressing genes crucial for the integrity of vascular structure. A decrease in the proportion of vSMC-like pericytes is observed with RCC progression, indicating that tumors may acquire the ability to invade and metastasize by disrupting vascular integrity. Furthermore, pericytes interact with other cells in the TME, including their transformation into myofibroblast-like cancer-associated fibroblasts (myCAFs), which promote the EMT process in RCC, their role in angiogenesis by interacting with endothelial cells, and their contribution to vascular dysfunction by altering the ECM, thereby facilitating RCC progression	Alchahin et al. [175] Davidson et al. [131] Zhang et al. [483] Du et al. [455]
Other stromal cells vSMCs	In RCC, mesenchymal cells can be categorized into multiple subpopulations, such as smooth muscle cells (SMCs), classic mesenchymal cells (MSG), and MSG.inf. MSG.inf characteristically expresses factors associated with stress and inflammation. In CDRCC, vascular smooth muscle cells (vSMCs) form a distinct cluster within the cellular population, marked by the specific expression of genes like *TPPP3*, *SPARCL1*, and *PDGFRB*. These vSMCs are believed to play a crucial role in maintaining microenvironmental stability and regulating the function of the tumor vasculature	Pan et al. [145] Davidson et al. [131]

## Clinical applications of single-cell sequencing technology in renal cancer

### Single-cell sequencing technology assists in renal cancer diagnosis

Accurate molecular classification and early diagnosis of RCC remain significant clinical challenges, particularly due to disease's complex heterogeneity. Traditional bulk sequencing approaches often mask critical molecular features, but scRNA-seq has emerged as a powerful tool to address these limitations and provide unprecedented resolution in tumor characterization.

Recent multi-omic analyses have further enhanced our understanding of RCC progression. Hu et al. [190] conducted comprehensive multi-omic profiling of ccRCC, identifying specific metabolic reprogramming patterns associated with disease progression. Their integrated analysis of transcriptomic, proteomic, and metabolomic data revealed distinct metabolic signatures that could serve as potential diagnostic markers. Additionally, Chen et al. [213] focused specifically on nccRCC through single-cell genomics, demonstrating that TME heterogeneity significantly correlates with clinical prognosis, providing new perspectives for molecular diagnosis of these less common RCC subtypes. Single-cell transcriptional analysis has revolutionized our understanding of RCC cellular origins. Young et al. [438] analyzed 1,300 childhood and adult kidney tumors using scRNA-seq, developing a quantitative approach to measure reference"cellular signals"in each tumor. They established cellular definitions of human renal tumors based on transcriptional profiles derived from single-cell mRNA reference maps of normal tissues. Their analysis revealed that childhood tumors consistently exhibit fetal cellular signals, providing a quantitative measure of immaturity. Most adult cancers lacked evidence of dedifferentiation towards a fetal state, contrary to prior hypotheses. Cases where transcriptional evidence of dedifferentiation was detected in adult tumors were associated with worse outcomes. Childhood tumors showed distinct differentiation trajectories, such as neural crest to mesenchyme conversion in malignant rhabdoid tumors, while adult tumors displayed transcriptional similarities to specific renal tubular cells, indicating distinct cellular origins and developmental pathways. Zhang et al. [122] further advanced the field through comprehensive single-cell transcriptomic profiling across multiple tumor types. Their analysis of 270,190 single-cell transcriptomes from 55 diverse samples revealed critical insights into tumor origins. The data demonstrated pronounced transcriptional similarities between PT cells and ccRCC, while chrRCC showed strong alignment with ICs. Validation through dual RNA in situ hybridization confirmed specific coexpression patterns of CA9/ITGB8 and CA9/ALPK2 in ccRCC tissues. The study delineated 31 distinct cellular populations, encompassing 12 epithelial, 8 immune, 6 stromal, and 5 EC states. Analysis of 23 established ccRCC driver genes revealed selective enrichment in PT cells, while newly identified markers ITGB8 and ALPK2 demonstrated remarkable diagnostic potential, achieving 92% sensitivity and 95% specificity in distinguishing ccRCC from other kidney tumors. This insight into cellular origins has significant implications for developing targeted therapies and identifying early diagnostic markers.

Differential diagnosis in morphologically similar tumors has been challenging, but Yu et al. [255] performed scRNA-seq and scATAC-seq on 19 ccRCC samples. Their analysis of 102,723 high-quality single-cell transcriptomes revealed comprehensive gene expression and DNA regulation information of ccRCC. Through integrated analysis of transcriptome and chromatin states, they identified specific regulatory features and discovered novel lncRNAs (RP11-661C8.2 and CTB-164N12.1) that promoted ccRCC invasion and migration, which was validated through in vitro experiments. Their approach provided new insights into the biology and potential treatment targets of ccRCC.

### Single-cell sequencing technology facilitates prognostic stratification

Despite advances in treatment options, accurately predicting disease progression and treatment outcomes remains a critical challenge in renal cancer management. Traditional prognostic models based on bulk tissue analysis often fail to capture the complex cellular interactions that influence patient outcomes. SCS has revolutionized our approach to prognostic stratification by revealing intricate relationships between cellular heterogeneity and clinical outcomes.

A fundamental challenge in prognostic assessment lies in understanding the evolution of immune dysfunction during disease progression. Braun et al. addressed this through a comprehensive single-cell profiling study of 164,722 cells from 13 patients across clinical stages [225]. Their analysis identified specific cellular transitions in CD8 + T cells, from cytotoxic to terminally exhausted states, with these exhausted cells being enriched in metastatic disease. These findings have immediate clinical applications, as their analysis showed the co-occurrence of terminally exhausted CD8 + T cells and M2-like macrophages was associated with worse prognosis in external cohorts. Saout et al. [256] performed single-cell RNA sequencing on ccRCC tumor samples, revealing varying degrees of expression-based intratumor heterogeneity (eITH) across tumors. They identified a unique tumor sample displaying high eITH with distinct malignant cell subpopulations exhibiting aggressive features related to EMT, angiogenesis, cell migration, and adhesion. Using transcriptomic signatures from these malignant cells, they developed a deconvolution-based strategy for risk stratification, potentially improving patient identification among those initially classified as low-risk. In the context of immunotherapy response prediction, Kinget et al. [328] conducted a comprehensive multiomics mapping of advanced RCC, identifying patient-specific crosstalk between pro-inflammatory tumor-associated macrophages and exhausted CD8 + T cells. They developed a tumor transcriptomic signature that correlated with positive outcomes following immune checkpoint blockade treatment, validated in real-world and independent clinical cohorts. Their findings highlight the potential of machine learning approaches for predicting immunotherapy response. Other studies have also identified spatial heterogeneity features in the tumor microenvironment [257].

Stromal components and their interactions with tumors have also been implicated in disease progression and immunotherapy response. Davidson et al. [131] identified specific mesenchymal-like tumor cells and myofibroblastic cancer-associated fibroblasts as key determinants of disease progression and immunotherapy response in ccRCC. Their findings suggest that stromal cell states could serve as valuable prognostic indicators. Li et al. [485] provided additional insights through detailed mapping of single-cell transcriptomes in both intratumoral and associated territories of kidney cancer, revealing spatial patterns of cellular interaction that correlate with disease outcomes. Furthermore, Shi et al. [176] focused on a particularly challenging aspect of RCC progression—vena caval tumor thrombus. Their single-cell analysis decoded the complex multicellular ecosystem within these structures, identifying distinct cellular compositions that could predict aggressive disease behavior.

For clinical implementation, Recent scRNA analyses have revealed significant cellular heterogeneity in RCC, with studies identifying distinct cell populations and their interactions within the tumor microenvironment [122].

### Single-cell sequencing technology predicts treatment response

Recent investigations have revealed specific molecular mechanisms underlying treatment response. Au et al. [486] conducted an extensive analysis of anti-PD-1 response and resistance in ccRCC, identifying key determinants of immunotherapy efficacy. Their findings highlighted specific molecular signatures associated with treatment outcomes, providing potential biomarkers for patient stratification. Aggen et al. [365] demonstrated that blocking IL1β can promote tumor regression while remodeling the myeloid compartment, offering new therapeutic strategies for RCC treatment. In a focused study on a specific RCC subtype, Dong et al. [242] investigated genomic characteristics and single-cell profiles in fumarate hydratase-deficient RCC following immunotherapy, revealing unique response patterns and potential therapeutic targets for this rare but aggressive variant.

Treatment resistance and variable therapeutic responses present major challenges in renal cancer management. Traditional methods of predicting treatment outcomes often fail to capture the complex cellular mechanisms underlying therapeutic response. scRNA technology has emerged as a powerful tool for understanding treatment resistance mechanisms and predicting therapeutic responses, particularly in the context of immunotherapy and targeted therapy. A fundamental challenge in prognostic assessment lies in understanding cellular mechanisms of treatment resistance. Building upon their previous findings, Braun et al. [225] further analyzed 164,722 cells across disease stages, revealing additional insights into treatment implications. They found that terminally exhausted CD8 + T cells were enriched in metastatic disease and showed restricted TCR diversity, with a newly identified bi-directional inhibitory circuit between these exhausted T cells and M2-like macrophages. The progressive loss of pro-inflammatory macrophages and concurrent increase in suppressive M2-like macrophages in advanced disease provided crucial insights for therapeutic targeting.

Understanding the dynamic changes during immunotherapy poses another significant challenge. Bi et al. [226] conducted single-cell transcriptome analysis of metastatic RCC before and after ICB exposure. They identified distinct response patterns: responders showed robust activation of cytotoxic T cell subsets with increased expression of co-inhibitory receptors and effector molecules. Their work revealed cancer cells bifurcating into two subpopulations after ICB treatment, with differential angiogenic signaling patterns and immunosuppressive programs. These findings provide critical markers for monitoring treatment efficacy.

The heterogeneous response to therapy presents a particular challenge in treatment planning. Zhang et al. [130] analyzed 37,243 single cells from seven ccRCC tumors and five matched normal samples, revealing critical insights into tumor heterogeneity and therapeutic targets. They identified that C4-C3AR1 + macrophages infiltration correlated with worse survival, while C3-CD163 + macrophages and C8-cDC2 infiltration predicted better outcomes. They also discovered IRX3 as a key transcription factor in tumor progression, with its nuclear localization specifically associated with metastatic sites. Furthermore, their analysis of ligand-receptor pairs, particularly C3-C3AR1 and LGALS9-CD47 interactions, provided new potential therapeutic targets for ccRCC. Real-time monitoring of treatment response remains a significant clinical challenge. Krishna et al. [487] conducted paired scRNA-seq and scTCR-seq of 167,283 cells from multiple tumor regions, lymph nodes, normal kidney, and peripheral blood from two ICB-naïve and four ICB-treated patients. Their analysis established detailed immune cell atlas of ccRCC, revealing extensive heterogeneity in immune infiltration patterns both within and between patients. Notably, they found distinctive immune landscapes associated with treatment response: ICB-responsive patients showed enrichment of CD8A + tissue-resident T cells, while resistant patients displayed elevated TAMs. Their TCR trajectory analysis framework revealed distinct T cell differentiation pathways between responders and non-responders to immunotherapy. Importantly, they found that pre-therapy levels of tissue-resident T cell signatures correlated with better response to ICB-based combination therapies across multiple independent cohorts. This framework provides critical insights for understanding treatment efficacy and potential therapeutic targeting. Zhang et al. [122] demonstrated through single-cell analysis that metastatic ccRCC patients with higher EC fractions showed poor response to nivolumab immunotherapy. This finding suggests TME composition, particularly EC proportion, may serve as a potential predictive biomarker for immunotherapy response.

Most recently, Yang et al. [366] made a significant breakthrough in identifying distinct populations of cytotoxic CD4 + CTLs associated with immunotherapy response. Notably, CD4 + T cells from immunotherapy responders exhibited high expression of cytotoxic-associated genes (such as GZMK and NR4A2), while non-responders'CD4 + T cells showed elevated levels of regulatory and inhibitory molecules (including FOXP3 and CTLA4). Analysis of the CheckMate 009 and 025 immunotherapy cohorts demonstrated that patients with higher CD4 + CTLs abundance showed significantly improved overall survival and PFS. This finding suggests the potential value of CD4 + CTLs as a biomarker for predicting immunotherapy response in mRCC patients.

### Clinical trials and treatment innovation

The translation of SCS insights into clinical practice represents a critical step toward personalized medicine in renal cancer treatment. Clinical trials investigating SCS applications have shown promising results in guiding therapeutic decisions and monitoring treatment responses. These trials span multiple aspects of renal cancer management, from biomarker validation to treatment strategy optimization.

In the context of immunotherapy selection, NCT04770246 is evaluating the utility of single-cell profiling in predicting response to ICB therapy. This trial specifically examines immune cell populations and their states before and during treatment, aiming to establish reliable predictive markers for immunotherapy response. Initial results suggest that specific T cell phenotypes identified through single-cell analysis may predict treatment outcomes with greater accuracy than conventional markers.

**Fig. 6:**
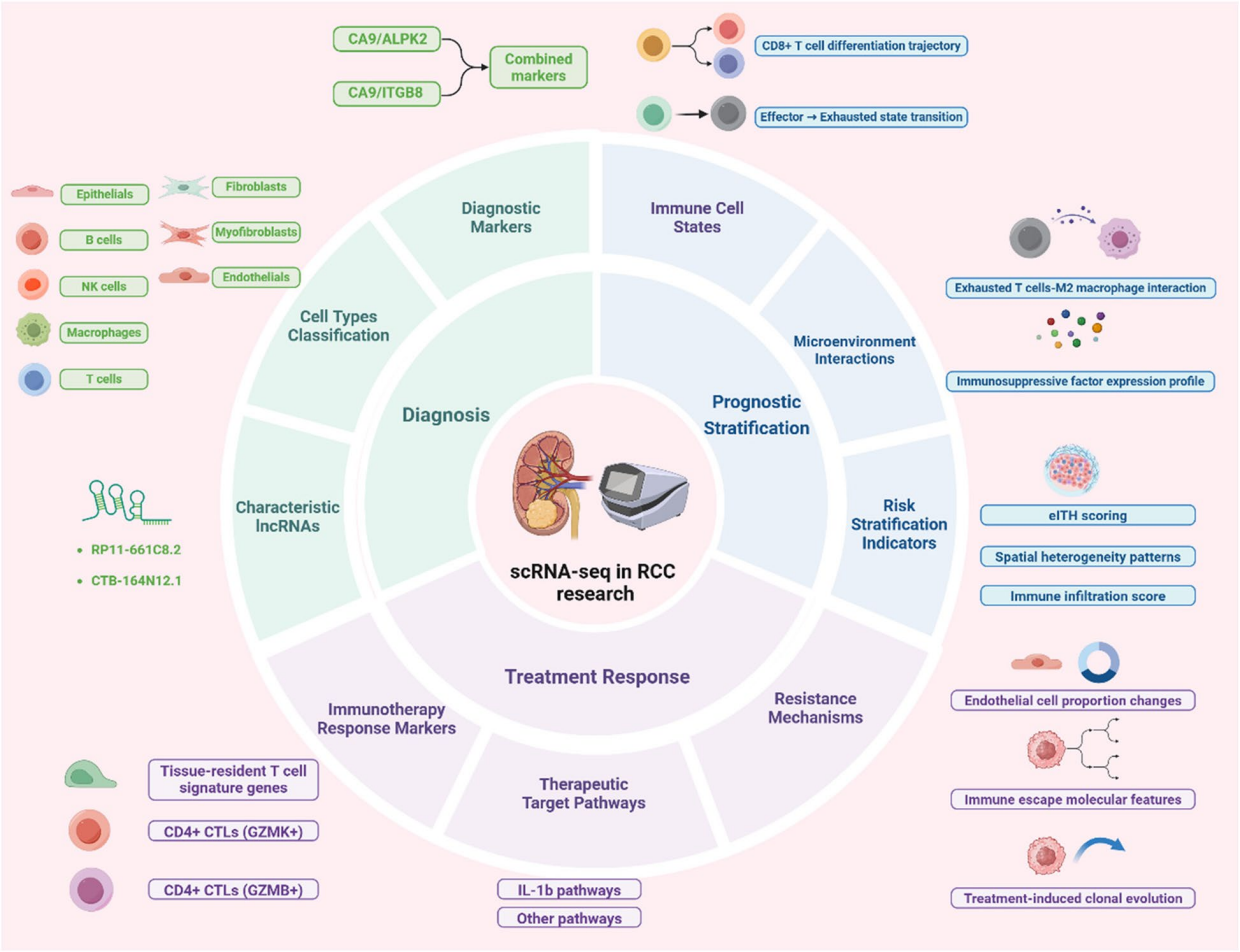
Applications of Single-cell Sequencing in Renal Cell Carcinoma Research. Single-cell sequencing analysis in RCC reveals three major research applications depicted in a radial layout. The inner circle represents the core technology application in RCC research. The middle layer illustrates three key research directions: diagnosis, focusing on cell-type identification and molecular markers (CA9/ITGB8, CA9/ALPK2); prognosis, highlighting immune cell states and risk assessment through eITH scoring; and treatment response, emphasizing immunotherapy markers and resistance mechanisms. The outer layer details specific cellular and molecular features, including PT-B cells in ccRCC, CD8+ T cell differentiation trajectories, and treatment-induced molecular alterations. This figure was created based on the tools provided by Figuredraw.com and Biorender.com

The BIONIKK trial (NCT02960906) represents another significant advance in biomarker-driven treatment selection. This study utilizes SCS to classify patients into molecular subgroups, guiding the choice between immunotherapy and targeted therapy. Preliminary data indicate that this approach may improve response rates compared to standard selection methods, with particular subgroups showing markedly better outcomes with specific treatment modalities. Applications of single-cell sequencing in renal cell carcinoma research are summarized in Fig. 6. Clinical trials implementing single-cell sequencing in renal cell carcinoma research are summarized in Table 2.

**Table 2:** Summary of clinical trials implementing single-cell sequencing in renal cell carcinoma research

NCT number	Phase	Title	Status	Primary outcome measures	Single-cell sequencing-related outcome	Enrollment
NCT05485896	Phase 2	A Prospective Single-arm Clinical Study of Pembrolizumab Combined with Lenvatinib Neoadjuvant Therapy in Patients With Advanced Renal Cell Carcinoma	Completed	1. Tumor responses evaluation using RECIST criteria. Adverse events monitoring according to CTCAE v5.0	Bulk RNA-seq and/or single cell RNA-seq were performed in part of tissues before and after neoadjuvant treatment to seek biomarkers predicting Response	23
NCT06195150	Observational Study	Overtaking Intra and Inter Tumoral Heterogeneity In Von Hippel-Lindau Related Renal Cancer (ITHORinVHL)	Recruiting	1. Integration of imaging and multiregion biopsy sampling. Determine immune and stromal heterogeneity 2. Generation and characterization of patient-derived organoids	Single cell RNA sequencing will be performed to analyze immune and stromal heterogeneity in VHL-ccRCC	3
NCT04495894	Phase 2	Pre-Incisional Ketorolac for Patients Undergoing Surgery for Non-Small Cell Lung Cancer and Renal Cell Carcinoma	Terminated	1. Incidence of blood transfusion. Incidence of hematoma development 2. Incidence of return to OR for bleeding. Incidence of postoperative renal failure 3. Incidence of postoperative morbidity	Single cell RNA sequencing will be performed to evaluate the effects of ketorolac on immune response pathways	82
ChiCTR2000030405	Phase 2	Study of Efficacy and Safety of Preoperative Axitinib Combined with Toripalimab in Renal Cell Carcinoma with Inferior Vena Cava Tumor Thrombus: a Clinical Multicentric Prospective Study	Prospective registration	The down-staging rate of IVC-TT at 6 weeks after treatment	Single cell RNA sequencing was performed to identify biomarkers of therapy effectiveness, which can guide strategy of neoadjuvant treatment	25

## Conclusion and future perspectives

SCS technology has significantly advanced renal cancer research. It has provided detailed insights into the cellular origins of various renal cancer subtypes, elucidating the potential roles of different cell types. SCS has also enhanced our understanding of the mechanisms underlying renal cancer initiation and progression, including EMT, metabolic reprogramming, immune evasion, gene expression regulation, and complex cell–cell communication networks. Moreover, SCS has illuminated the complex TME, demonstrating the heterogeneity and functions of immune cells. In clinical applications, SCS has demonstrated potential in renal cancer diagnosis by identifying novel biomarkers and improving molecular classification. It has facilitated prognostic stratification by elucidating the relationships between cellular heterogeneity and clinical outcomes, and has contributed to predicting treatment response, particularly in immunotherapy and targeted therapy. Clinical trials based on SCS are currently in progress, aiming to translate these findings into personalized medicine for renal cancer treatment.

Despite the advancements in SCS technology, significant challenges persist in data processing complexity and associated high costs. Future research endeavors should prioritize the development of more efficient computational methods and experimental designs to mitigate costs and enhance the accuracy and efficiency of data analysis [488, 489]. For instance, further optimization of algorithms for single-cell multi-omics data integration and improvement of clustering and classification algorithms'performance are necessary. Enhancing the resolution and sensitivity of SCS techniques to detect rare cell populations and low-abundance transcripts or proteins could provide a more comprehensive understanding of the TME. This may necessitate the development of novel sequencing platforms or the refinement of existing ones. Investigating the dynamic changes of the TME during cancer progression and treatment is crucial. Longitudinal studies utilizing SCS could track the evolution of different cell types and their interactions over time, which would elucidate the mechanisms of tumor recurrence and metastasis and identify potential therapeutic targets at various stages of the disease. A more detailed elucidation of epigenetic and transcriptional regulatory networks could uncover innovative therapeutic approaches. Specifically, investigating how TFs interact with epigenetic modifiers in renal carcinoma cells and their tumor microenvironment may enable targeted drug development against these coordinated regulatory mechanisms. Integrating SCS with other clinical data, such as patient demographics, imaging data, and treatment history, could enhance the predictive power of prognostic models and treatment response prediction [490]. This multi-modal approach may provide a more comprehensive view of the patient's disease status and inform more personalized treatment decisions. Validating the identified biomarkers in larger and more diverse patient cohorts is necessary to ensure their clinical utility. Additionally, developing standardized protocols for SCS sample collection, processing, and analysis would enhance the reproducibility and comparability of research results across different studies. Exploring the potential of SCS in monitoring treatment response in real-time could enable early detection of treatment failure or resistance, allowing for timely adjustment of treatment strategies. This may involve the development of non-invasive or minimally invasive methods for obtaining single-cell samples during treatment.

In conclusion, while SCS has made significant progress in renal cancer research, continued efforts in technology development, biological exploration, and clinical translation are essential to fully realize its potential in improving renal cancer diagnosis, prognosis, and treatment.

## Data Availability

No datasets were generated or analysed during the current study.

## Abbreviations

ccRCC: Clear cell renal cell carcinoma
pRCC: Papillary renal cell carcinoma
chrRCC: Chromophobe renal cell carcinoma
TRCC: Translocation renal cell carcinoma
ESCRCC: Eosinophilic solid and cystic renal cell carcinoma
RO: Renal oncocytoma
AML: Angiomyolipoma
HLRCC: Hereditary leiomyomatosis and renal cell carcinoma
BHD-RCCs: Birt-Hogg-Dubé-associated kidney cancer
VHL-RCC: Von Hippel-Lindau-associated kidney cancer
s-RCC: Sporadic clear cell renal cell carcinoma
RMC: Renal medullary carcinoma
CDRCC: Collecting duct renal cell carcinoma
PT: Proximal tubule
vSMCs: Vascular smooth muscle cells
ICs: Intercalated cells
EGF: Epidermal growth factor
EGFR: Epidermal growth factor receptor
VCAM1: Vascular Cell Adhesion Molecule 1
PST: Proximal straight tubules
CSCs: Cancer stem cells
MCs: Malignant cells
SIX2: Sine oculis homeobox 2
CITED1: Cbp/p300-interacting transactivator with Glu/Asp-rich carboxy-terminal domain 1
TAL: Thick ascending limb
MPs: Meta-programs
EMT: Epithelial-mesenchymal transition
NMF: Non-negative matrix factorization
CNs: Cellular niches
T: Tumor core
IM: Invasive margin
SN: Normal region
TAGLN: Transgelin
KRT19: Keratin 19
SOD2: Superoxide dismutase
MALAT1: Metastasis-associated lung adenocarcinoma transcript 1
RHPs: Recurrent heterogeneity programs
*FN1*: Fibronectin
*VIM*: Vimentin
*JUP*: Plakoglobin
OCLR: One-class logistic regression
ccRCC.mes: Mesenchymal-like ccRCC cells
myCAFs: Myofibroblast-like cancer associated fibroblasts
ECM: Extracellular matrix
TFs: Transcription factors
SNAIL: Snail family transcriptional repressor
TWIST: Twist basic helix-loop-helix transcription factor
BMRCC: Bone metastatic RCC
EMP1: Epithelial membrane protein 1
COL3A1: Collagen type III alpha 1 chain
WNT: Wingless-type MMTV integration site family
SIRPα: Signal regulatory protein alpha chain
CD47: Integrin-associated protein
PT: Primary tumor
TT: Tumor thrombus
nccRCC: Non-clear cell renal cell carcinoma
TFCP2L1: Transcription factor CP2-like 1
MYC: Myelocytomatosis oncogene
DCCD: De-clear cell differentiation
GSH: Glutathione
GSSG: Glutathione disulfide
ROS: Reactive oxygen species
FAO: Fatty acid oxidation
Cu: Copper
CuCOX: Cytochrome c oxidase
OXPHOS: Oxidative phosphorylation
PPAR: Peroxisome proliferator-activated receptors
CAFs: Cancer-associated fibroblasts
TME: Tumor microenvironment
EPCs: Endothelial progenitor cells
TIME: Tumor immune microenvironment
NNMT: Nicotinamide-N-methyltransferase
wGII: Weighted gene instability index
PYCR1: Pyrroline-5-Carboxylate Reductase-1
PVR: Poliovirus receptor
LGALS9: Galectin-9
FHRCC: Fumarate hydratase-deficient renal cell carcinoma
PFS: Progression-free survival
TRM: Tissue-resident memory T
PD-L1: Programmed cell death ligand 1
NECTIN2: Nectin cell adhesion molecule 2
MIF: Macrophage migration inhibitory factor
CSF1: Colony-stimulating factor-1
ICB: Immune chekpoint blockade
TAMs: Tumor-associated macrophages
TIM-3: T cell immunoglobulin and mucin domain 3
TIGIT: T-cell immunoglobulin and immunoreceptor tyrosine-based inhibitory motif domain
SPP1: Secreted phosphoprotein 1
TREM2: Triggering receptor expressed on myeloid cells 2
APOE: Apolipoprotein E
C1Q: Complement Component 1, Q subcomponent
ITGB1: Integrin β1
ITGA4: Integrin α4
CTL: Cytotoxic T lymphocyte
Tregs: Regulatory T cells
PLOD2: Procollagen-lysine 2-oxoglutarate 5-dioxygenase 2
SAA1: Serum amyloid A 1
LIGHT: Lymphotoxin-like inducible protein that competes with glycoprotein D for herpesvirus entry mediator on T cells
CXCR4: C-X-C motif chemokine receptor 4
LRP: Low density lipoprotein receptor-related protein
C3: Complement component 3
C3AR1: C3a receptor 1
ECs: Endothelial cells
PILRα: Paired immunoglobulin-like type 2 receptor α
PR: Partial response
PD: Progressive disease
Fas: Factor associated suicide
SLC38A8: Solute carrier family 38 member 8
ACRs: Chromatin regions
MAFA: Musculoaponeurotic fibrosarcoma oncogene homolog A
TUBB3: Class III β-tubulin
R: Receptor
TGF-β: Transforming growth factor – β
GAS6: Growth arrest-specific protein 6
AXL: AXL receptor tyrosine kinase
PDK: Pyruvate dehydrogenase kinase
MPZ: Myelin protein zero
ANG: Angiogenin
PLXNB2: Plexin-B2
BMP: Bone morphogenetic protein
VEGFA: Vascular endothelial growth factor A
EGLN3: Egl-9 family hypoxia-inducible factor 3
GATA6: Gata—binding protein 6
GDF15: Growth differentiation factor 15
TERT: Telomerase reverse transcriptase
CRC: Colorectal cancer
YAP: Yes—associated protein
TAZ: Transcriptional co—activator with PDZ—binding motif
AHNAK: Desmoyokin
HIF1α: Hypoxia—inducible factor 1—α
lncRNAs: Long non-coding RNAs
HPSE2: Heparanase 2
ATRNL1: Attractin—like 1
IGSF21: Immunoglobulin superfamily member 21
CNVs: Copy number variations
CXCL: C-X-C motif chemokine
CCL: C—C motif chemokine ligand
ENL: AF9 family transcriptional elongation regulator
Fox: Forkhead box
AP-1: Activator protein—1
FOS: FBJ murine osteosarcoma viral oncogene homolog
FOSL2: FOS—like antigen 2
c-FOS: Cellular-FOS
JUN: Jun proto—oncogene
c-JUN: Cellular-JUN
CA9: Carbonic anhydrase IX
TNF: Tumor necrosis factor
MAPK: Mitogen-activated protein kinase
OTP: Orthopedia
VENTX: Ventral anterior homeobox
HOXC5: Homeobox C5
ISL1: Insulin gene enhancer binding protein 1
FXYD2: FXYD domain-containing ion-transport regulator 2
CRYAB: Crystallin alpha B
ZEB1: Zinc finger E-box-binding homeobox
SFFV proviral integration oncogene spi-C: Spleen-focus-forming virus
EOMES: Eomesodermin
ETV4: ETS variant transcription factor 4
ETS transcription factor 1: E-twenty-six
EBF2: Early B-cell factor 2
SOX8: SRY-related HMG-box 8
HNF: Hepatocyte nuclear factor
TEAD3: TEA domain transcription factor 3
NFIB: Nuclear factor I B
CEBPB: CCAAT/enhancer-binding protein beta
KLF: Kruppel-like factor
NR1H4: Nuclear receptor subfamily 1 group H member 4
RARA: Retinoic acid receptor alpha
STAT3: Signal transducer and activator of transcription 3
NFκB: Nuclear factor kappa-light-chain-enhancer of activated B cells
POU2F2: POU domain, class 2, transcription factor 2
TR Mac: Tissue-resident macrophages
IL: Interleukin
bZIP TF: Basic-leucine zipper
MMPs: Matrix metalloproteinases
LTB: Secrete lymphotoxin beta
LTBR: Lymphotoxin beta receptor
FasLG: Fas ligand
VHL: Von Hippel-Lindau
GZM: Granzyme
ACVR: Activin A receptor
FGF: Fibroblast growth factor
COL1A1: Collagen type I alpha 1
DDR1: Discoidin domain receptor 1
HMMR: Hyaluronan-mediated motility receptor
INHBA: Inhibin beta A
LTF: Lactotransferrin
HG: High-grade
LG: Low-grade
PD-1: Programmed cell death protein 1
LAG3: Lymphocyte-activation gene 3
TCR: T-cell receptor
TCRB: T cell receptor beta chain
KLRG1: Killer cell lectin-like receptor G1
GNLY: Granulysin
CTLA4: Cytotoxic T-lymphocyte-associated protein 4
HAVCR2: Hepatitis A virus cellular receptor 2
ST-seq: Spatial transcriptomics sequencing
TCF7: Transcription factor 7
ENTPD1: Ectonucleoside triphosphate diphosphohydrolase 1
BATF: Basic leucine zipper transcription factor ATF-like
TOX: Thymocyte selection-associated high mobility group box protein
PRDM1: Positive regulatory domain zinc finger protein 1
NR4A1: Nuclear receptor subfamily 4 group A member 1
NFAT: Nuclear factor of activated T – cells
cDC1: Conventional type 1 dendritic cell
sarRCC: Sarcomatoid RCC
RCCpm: RCC pancreatic metastasis
PAX8: Paired box gene 8
HLA: Human leukocyte antigen
MX1: Myxovirus resistance 1
4-1BB-Lo: 4-1BB ligand-low expression
GRB: Growth-related oncogene B
IM: Immunological subtypes
scNanoRNAseq: Single-cell long-read sequencing
DCIs: Differential expression of transcript isoforms
MDTs: Most dominant transcripts
IEG: Immediate early gene
SELL: Selectin L
LEF1: Lymphoid enhancer-binding factor 1
TFH: Follicular helper T cells
TNFRSF: Tumor necrosis factor receptor superfamily
STR: Stress-reactive
HSPA1: Heat shock protein family A member 1
MECRGs: Malignant epithelial cell-related genes
TI Treg: Tumor-infiltrating Treg
PB: Peripheral blood
CF1: Cell Fate #1
CF2: Cell Fate #2
ART: Normal renal tissue
CR: Chemokine receptor
mRCC: Metastatic RCC
RCN1: Reticulocalbin-1
TKIs: Tyrosine kinase inhibitors
ISG: Interferon-stimulated gene
IRF: Interferon regulatory factor
DCs: Dendritic cells
FGL1: Fibrinogen-like protein 1
MSR1: Macrophage scavenger receptor 1
CSF1R: Colony-stimulating factor 1 receptor
C1QC: C1q c chain
SPP1: Selenoprotein P plasma 1
FOLR2: Folate receptor 2
S100A: S100 calcium-binding protein A
MHC: Major histocompatibility complex
TAM ISGint, anti-tumor: Intermediate interferon signaling gene expression
TAM HLAhi: High HLA-DR expression
pro-tumorigenic: Tissue-resident monocytes
CTSS: Cathepsin s
LGALS3: Lectin galactoside-binding soluble 3
RGCC: Regulator of cell cycle
MEF: Myocyte-enhancer factor
TCF7L2: TCF7 ligand 2
IGF: Insulin-like growth factor
THBS1: Thrombospondin 1
NDUFA4L2: NADH dehydrogenase [ubiquinone] 1 alpha subcomplex 4—like 2
NK: Natural killer
XCL: Chemokine (C - motif) ligand
KLR: Killer-cell lectin-like receptor subfamily
FGFBP2: FGF-binding protein 2
CX3CR1: C-X3-C chemokine receptor 1
NCAM: Neural cell adhesion molecule
FCGR3A: Fc fragment of igg receptor III a
dim: Dimly expressed
hi: Highly expressed
LAMP: Lysosome—associated membrane protein
EGR: Early growth response gene
CAPG: Capping protein gelsolin – like
PRF: Prolactin—releasing factor
NCR: Natural cytotoxicity triggering receptor
mDCs: Myeloid dendritic cells
pDCs: Plasmacytoid DCs
FLT: Fms-like tyrosine kinase
CLEC9A: C-type lectin domain family 9 member A
CCR: C-C chemokine receptor (A cell-surface protein that binds chemokines to guide immune cell movement)
LILR: Leukocyte immunoglobulin-like receptor
LYPD3: Ly6/PLAUR domain-containing proteins 3
FZD: Frizzled class receptor
MT: Metallothionein
MS4A1: Membrane-spanning 4 – domains subfamily A member 1
TAABs: Tumor-associated atypical B cells
Bgc: Germinal center B cells
IGHG1: Immunoglobulin heavy chain gamma 1
KIT: Kinase insert domain receptor
TPSAB1: Tryptase alpha/beta 1
ADGR: Adhesion G protein-coupled receptor
IGFBP: IGF-binding protein
TECs: Tumor-associated endothelial cells
KDR: Kinase insert domain receptor
ACKR: Atypical chemokine receptor
SERPINE2: Serine proteinase inhibitor clade E member 2
SOST: Sclerostin
KCNE3: Potassium voltage-gated channel subfamily E regulatory subunit 3
VWF: Von willebrand factor
ENPP: Ectonucleotide pyrophosphatase
EDNR: Endothelin receptor type
HSPG: Heparan sulfate proteoglycan
AVR: Ascending vasa recta
CHRBP: Chromatin-remodeling binding protein
POSTN: Periostin
deMuts: Differential mutations
RPS2: Ribosomal protein S2
PECAM: Endothelial cell adhesion molecule
CDH: Cadherin
ACTA2: Actin alpha 2 smooth muscle aorta
PDGFRB: Platelet-derived growth factor receptor beta
GJA5: Gap junction protein alpha 5
LY6H: Lymphocyte antigen 6 complex locus H
PGF: Placental growth factor
LOX: Lysyl oxidase
CHST1: Carbohydrate sulfotransferase 1
PXDN: Peroxidasin
SPARCL: Secreted protein acidic and rich in cysteine – like
CRHBP: Corticotropin-releasing hormone-binding protein
SMA: Smooth muscle actin
FABP: Fatty-acid-binding protein
iCAFs: Inflammatory
apCAFs: Antigen-presenting
XBP: X-box-binding protein
ATF: Activating transcription factor
FAP: Fibroblast activation protein
TGFBI: Transforming growth factor beta induced
THY: Thyroid-stimulating hormone
reRCC: Recurrent RCC
PLK: Polo-like kinase
HMGA: High-mobility group A
PTTG: Pituitary tumor-transforming gene
MPZL1: MPZ like-1
TIMP: Tissue inhibitor of metalloproteinase
RERGL: Ras-related and estrogen-regulated growth inhibitor–like
MCAM: Melanoma cell adhesion molecule
SMCs: Smooth muscle cells
MSG: Mesangial cells
REN: Renin
ANX: Annexin
FPR: Formyl peptide receptor
SCS: Single-cell sequencing
FACS: Fluorescence-Activated Cell Sorting
cDNA: Complementary DNA
scRNA-seq: Single-cell RNA sequencing
scATAC-seq: Single-cell chromatin accessibility
scMetabolomics: Single-cell metabolomics
scProteomics: Single-cell proteomics
snRNA-seq sequencing: Single-nucleus RNA
TPPP3: Tubulin polymerization promoting protein family member 3
scTCR-seq: Single-cell T cell receptor sequencing
scBCR-seq: Single-cell B cell receptor sequencing
JAG: Jagged
LILRB: Leukocyte immunoglobulin-like receptor B
